# The association between age and medication-related hospital admission in adults: a systematic review and meta-analysis

**DOI:** 10.1007/s11096-026-02167-3

**Published:** 2026-06-04

**Authors:** Reema Mohammad Munshi, Eimear Ni Sheachnasaigh, Monica Strugaru, Folasade Olawoye, Catherine Comiskey, Tamasine Grimes

**Affiliations:** 1 Department of Pharmacy Practice, College of Pharmacy, Umm Al-Qura University, Makkah, Saudi Arabia; 2 School of Pharmacy and Pharmaceutical Sciences, Panoz Institute, Trinity College Dublin, The University of Dublin, Dublin, Ireland; 3 School of Nursing and Midwifery, Trinity College Dublin, The University of Dublin, Dublin, Ireland; 4 The National Rehabilitation Hospital, Dublin, Ireland

**Keywords:** Drug-related side effects and adverse reactions, Hospitalization, Risk factors, Systematic review

## Abstract

**Introduction:**

Medication-related hospital admission is a major contributor to preventable patient harm and healthcare burden worldwide. The relationship between age and medication-related hospital admission has not been comprehensively synthesised.

**Aim:**

To evaluate the association between age and medication-related admission in adults and to provide research recommendations.

**Method:**

MEDLINE (Ovid), EMBASE, Scopus, Web of Science, PsycINFO, CINAHL, Cochrane Library, and Global Health databases were searched from January 2000 to March 2025. Studies of adults reporting medication-related admission, providing data on participant age for an exposed and comparator group, published in English were included. Studies restricted to children were excluded. Selection through dual, independent title/abstract screening and full-text assessment using predefined eligibility criteria. Disagreements resolved by third reviewer or consensus. Study quality assessed using Joanna Briggs Institute tool for systematic reviews addressing questions of prevalence. Findings were reported descriptively and two meta-analyses were performed. Risk ratios of experiencing medication-related admission by age group (16–64 years-old, 65+years-old) were pooled. Mean ages of those experiencing medication-related admission compared to another cause of admission were pooled. Prediction intervals and sub-group analyses were used to explain heterogeneity.

**Results:**

Fifty studies, recruiting 210,514 participants, were included, with the majority judged as good quality. Thirteen studies exclusively recruited an older cohort, 30 studies were undertaken in Europe. Age data were variably described. The pooled risk ratio 1.64, (95% CI 1.15–2.35, K = 12, I^2^ = 96%, n = 56,101) suggested adults ≥ 65 years had a higher risk of experiencing medication-related admission than younger adults (16–64 years). Studies undertaken in Africa had a higher effect size. The pooled mean difference (2.86 years, 95% CI − 1.63–7.36; *p* = 0.21, K = 10, I^2^ = 93%, n = 12,440) indicated no statistically significant difference in age between groups. Risk of bias judgement was a statistically significant contributor to heterogeneity. Prediction intervals for both meta-analyses were wide, suggesting that findings of future studies could differ significantly. The certainty of evidence, GRADE approach, was very low.

**Conclusion:**

Age is an unreliable predictor for medication-related admission without accounting for confounding and context. Future research should consistently define age categories and better explore other risk factors for medication-related admission.

**Supplementary Information:**

The online version contains supplementary material available at 10.1007/s11096-026-02167-3.

## Impacts statements

- Medication related hospital admission is a global public health concern and understanding the associated risk factors is important.
- To better understand the association between age and medication-related hospital admission, future studies should use standardised age categorisations and employ robust multivariate statistical analysis adjusting for confounders.
- Future studies should explore the geographic variation in prevalence and risk factors for medication-related hospital admission.

## Introduction

Hospital admissions resulting from medication-related problems are prevalent and contribute to substantial health and financial burdens, impacting both patients and healthcare systems [1–3]. The median prevalence rates of hospitalization resulting from adverse drug reaction (ADR), adverse drug event (ADE) and other medication-related problems in adults have been estimated as 7%, 4.6% and 12.1%, respectively [4]. The median prevalence rate of hospital admissions, across all age groups, associated with medication non-adherence is estimated as 4.29% [5]. Medication-related hospitalisation is a global concern [6–9].

Theoretically, age-related alterations in pharmacokinetics and pharmacodynamics, deterioration in renal and hepatic function and an amplified sensitivity to several pharmacological classes can contribute to an increased risk of medication-related admission as people grow older [10]. An American study, 2011, reported that ADE-related hospitalisation was 3.5 times as high among adults 85 years of age or older and 65–69 years of age, although it did not adjust for the baseline hospitalisation rate of any age group [11]. It is widely reported that increasing age is a risk factor for medication-related admission and interventions to identify or manage such admissions have been designed accordingly [2, 6, 12]. However, other studies of medication-related harm as a cause of hospital admission have not identified advancing age as a risk factor [13, 14]. Reviews of medication-related admission have non-specifically reported age as a risk factor [4, 15].

Systematic review findings suggest a predominance of older populations in studies examining tools to identify medication-related hospital admissions [16, 17]. In addition, current relevant studies often explore the problem exclusively in older populations [3], including several large, European Union funded studies [18, 19]. These restrictions limit the potential to understand the prevalence, impact or risk factors for medication-related hospitalisation across the wider adult population. In the context of increased prevalence of polypharmacy in younger or middle-aged adults, strained healthcare resources and increased life expectancy globally, a more robust appreciation of the association between age and medication-related admission is urgently needed [20, 21]. A preliminary literature search identified no systematic review that considered the statistical association between medication-related admission and age.

## Aim

This review aimed to evaluate the association between age and medication-related admission and to provide research recommendations.

## Method

This review followed the Joanna Briggs Institute (JBI) guidelines for conducting and reporting systematic reviews of association (etiology) [22]. It was reported according to the Preferred Reporting Items for Systematic Reviews (PRISMA) statement [23], and was registered in the prospective register of systematic reviews in October 2018 (PROSPERO, CRD42019111598).

### Eligibility criteria and study selection

Eligibility criteria were explicitly structured using the Population, Exposure, Outcome framework, because this review was concerned with summarising a statistical association:[24, 25] 

*Participants*: Studies of adults experiencing hospital admission were eligible. For studies that recruited adults and children, only data for adults were considered in this review. Studies recruiting cohorts experiencing specific diseases, conditions, medication(s), poisonings, drug abuse or misuse were excluded. Studies that reported emergency department visit without hospital admissions were excluded. Adults were considered aged 16 years or older, because this is a common age of medical consent internationally.

*Exposure*: Studies that reported age data for a study group experiencing medication-related admission and a comparator group, henceforth referred to as non-medication-related admission, were eligible.

*Outcomes*: Studies that reported hospital admission caused or contributed by medication-related harm, including ADE, ADR, medication error, and medication non-adherence, regardless of how the study defined medication-related admission, were considered. The World Health Organization define medication-related harm as the harm caused by medication if taken incorrectly, monitored insufficiently, or as the result of an error, accident or communication problem [26, 27]. We operationalised this definition to group any admission caused by medication-related harm, including an ADE, ADR, medication error or medication non-adherence. Eligible studies needed to report the causality of medication-related problems causing hospital admission (as distinct from the causality of a medication causing the medication-related problem itself).

*Study design and characteristics*: The only no restriction on study design was that quantitative studies were included. Literature reviews, grey literature, conference proceedings, opinion pieces and commentaries, and qualitative studies were excluded. Studies published in any language other than English or prior to 2000 were excluded. This date restriction was applied because the report “To Err is Human” was published in 1999, setting a point in time for greater expected scrutiny on medication safety, including medication-related hospitalisation [28].

### Data sources and search strategy

Eight databases (MEDLINE (Ovid), EMBASE, Scopus, Web of Science, PsycINFO, Cumulated Index to Nursing and Allied Health Literature (CINAHL), Cochrane Library and Global Health) were searched from the year 2000 to March 2025. The search explored three concepts: (1) Medication-related problems, (2) Hospitalisation, and (3) Incidence or prevalence (Supplementary material, Search strings). The search strategy was adapted to the syntax and operability requirements of each database. Backward citation chasing was performed by reviewing the bibliography of each included study, and forward citation chasing was performed using Google Scholar.

Outputs from all searches were imported into EndNote version 9 (Clarivate Analytics, PA, USA) and de-duplicated before being imported into Covidence ® to support further de-duplication and data management.

### Study selection

All available records were screened independently first at the title and abstract stage, and then the full text stage by two reviewers selected from a team of four (RM, TG, EN, MS). The independent decisions at each stage were compared and discussed. Disagreements were resolved by discussion between the two reviewers (RM, TG, EN, MS), or by a third reviewer’s arbitration.

### Data extraction

Data were extracted against a template structured by the Population, Exposure, Outcome (PEO) framework:[24, 25]

- *Study characteristics*: Authors, publication year, study title, study period, geographic location, sample size, study design, hospital setting, and study participant eligibility criteria.
- *Participant characteristics (Population)*: Demographics: participant age, sex, polypharmacy and comorbidity for both the medication-related admission and non- medication-related admission groups.
- *Exposure characteristics (Exposure)*: Reported age measures (categorical or continuous) for the overall study population and separately for both groups, and the statistical methods used for age comparison.
- *Outcome characteristics (Outcome)*: Medication-related admission definition(s) used, type(s) and proportion of medication-related admissions, preventability and severity assessments employed and findings, assessment(s) applied to determine the strength of causality of (1) the medication-related problem causing the hospital admission and (2) the drug causing the medication-related problem, and the findings.

Data were extracted independently by two reviewers (RM, TG, EN, MS). Discrepancies were reviewed by a third author or resolved through consensus. Where required, corresponding authors were contacted to request missing or additional data that were unpublished in original articles.

### Assessment of quality and certainty of evidence

The methodological quality of included studies was assessed independently by two reviewers (RM, TG, EN, MS) using the Johanna Briggs Institute (JBI) standardised critical appraisal instrument for prevalence data for systematic reviews [29]. This checklist includes ten questions evaluating the study’s internal and external validity. The assessment facilitates a qualitative, domains-based rather than quantitative assessment of studies. The judgements for each domain within each study were visually appraised to generate an overall qualitative assessment of quality. Disagreements were resolved by discussion between two reviewers (RM, TG, EN, MS), or with a third reviewer. All included studies were analysed, regardless of quality.

The overall certainty of evidence was evaluated for each meta-analysed outcome using the GRADE approach, a systematic and transparent framework commonly applied in clinical research and guideline development. This approach extends beyond basic critical appraisal by assessing the consistency, robustness, and overall reliability of the available evidence. Through this evaluation, the strength of the evidence can be determined, whether it is strong, limited, or mixed. According to this framework, evidence is categorized into four levels of certainty: high, moderate, low, and very low [30].

### Data analysis

The extracted data were described in tables and narrative summaries, and meta-analysed, where possible.

#### Descriptive synthesis

The proportion of hospital admissions due to a medication-related problem was calculated as the number of patients admitted to hospital due to at least one medication-related problem divided by the total number of eligible patients in each study. The studies’ reported approaches to comparing age between study groups were summarised.

#### Meta-analysis

Two meta- analyses were undertaken using the metafor library in R Package:

1. Compared the likelihood of experiencing medication-related admission, relative to another cause for admission, for participants aged 16–64 years versus those aged ≥ 65-years. For each study, the relative risk (RR) with 95% confidence interval (CI) was calculated, and a pooled RR (95% CI) was calculated using a random-effects model. The count data in this model were unadjusted. A sensitivity analysis was performed by omitting two studies, one reported an outlying result and both recruited some participants younger than 16 years old, despite reporting an adult study population.
2. compared participant mean age by study group. For studies that reported mean age, the effect size of the mean age difference (MD) with 95% CI was calculated, and a pooled estimate was obtained using a random-effects model. For continuous age data, mean differences were pooled using study-reported group means, standard deviation (SD), and sample sizes for each group. The mean ages were unadjusted.

For both meta-analyses, heterogeneity between included studies was assessed using Cochran’s Q test and the I^2^ statistic [31]. Because this was identified as high, a random-effects model was used. Prediction intervals were calculated and subgroup analysis was performed to understand potential heterogeneity sources, and meta-regression was performed, where possible, to help explain the heterogeneity and account for between study variability in effect size. Funnel plots were visually inspected for evidence of between study heterogeneity and publication bias. Meta-analyses were conducted using R-package, library metafor, and forest plots were generated in Review Manager RevMan 5 (Copenhagen: The Nordic Cochrane Centre, Cochrane).

## Results

### Search and study selection

**Fig. 1:**
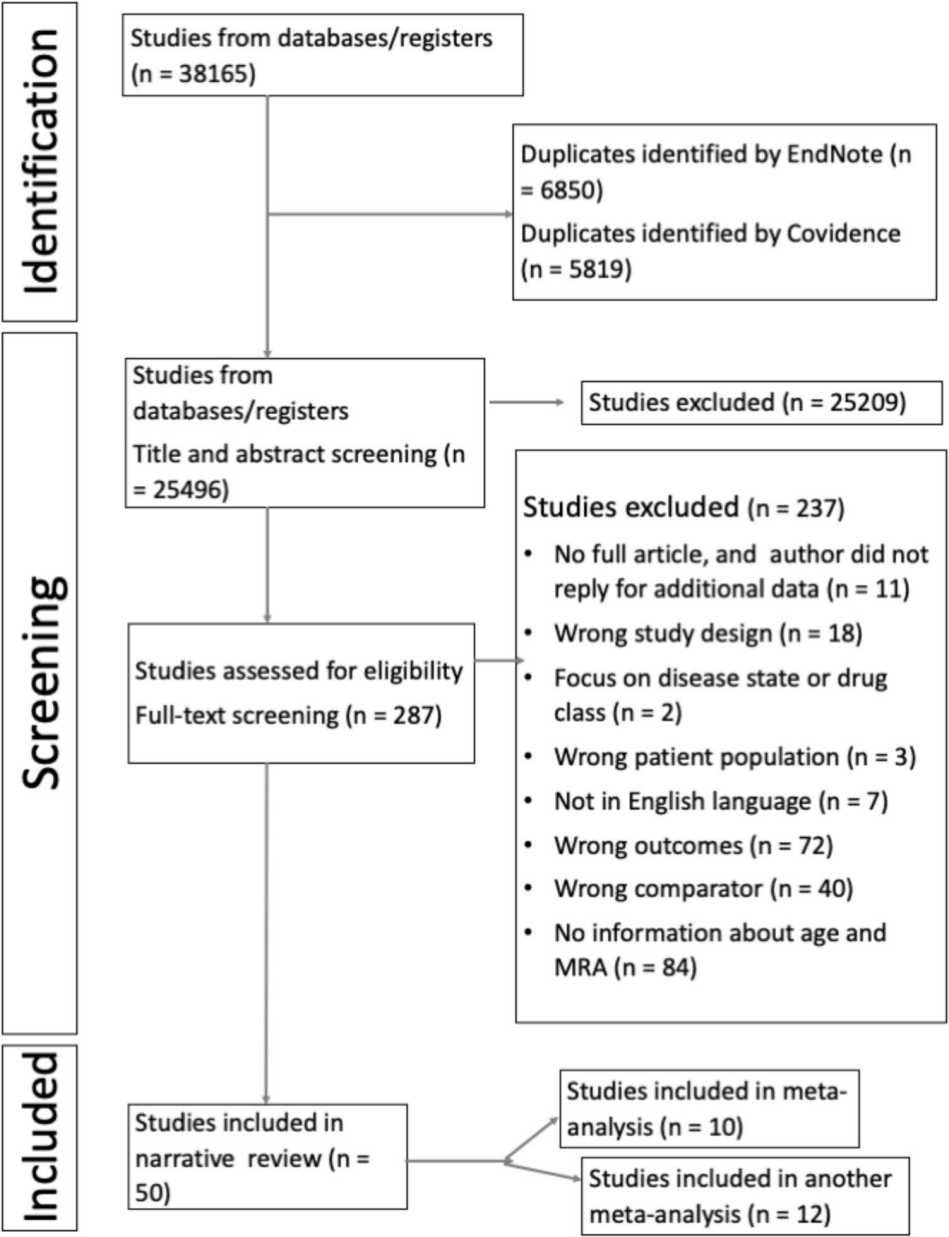
Flow diagram of the study selection process

From all sources, 38,165 studies were identified. After de-duplication, 25,496 studies were screened by title and abstract screening, 25,209 were excluded. Two-hundred-and-eighty-seven studies were subjected to full-text screening, with 237 excluded with reasons (Fig. 1). Fifty studies examining data for 210,514 participants were included in this review [32].

### Study characteristics

The majority of studies were cross-sectional (n = 33), followed by cohort (n = 12), case–control (n = 3), and pilot studies (n = 2). Study sample size ranged from 100 to 60,263 participants (Table 1). Seventeen studies were published between 2000 and 2009, 22 between 2010 and 2020, and 11 since 2021. Geographically, 30 studies (63%) were conducted in Europe. The remainder were undertaken in Africa (n = 5), South-east Asia (n = 3), Western Pacific (n = 4), North America (n = 3), South America (n = 1) or the Eastern Mediterranean region (n = 1).Three studies did not report their geographical location, (Supplementary material, Expanded characteristics).

**Table 1:** Characteristics of the 50 included studies

Author (year)	Study design	Stage when MRA was assessed/ hospital setting	Study sample size	Study population	Type of MRA	Number of patients with MRA	Prevalence of MRA %, 95% CI	Definition of medication-related problems
Adedapo [33]	P, C	HA, DH/M	1280	13 +	ADR	51	3.9, NA	WHO
Ahern [34]	P, CS	HA/E	856	A	ADR	75	8.8, 7.0–10.9	WHO
Alexopoulou [35]	P, CS	HA/E, M	548	15 +	ADR	70	12.8, NA	NR
Alvarez [36]	P, CS	HA/M	1045	–	ADR	112	10.7, 8.8–12.6	Own definition
Angamo [37]	P, CS	HA/M	1001	18 +	ADR	103	10.3, NA	WHO
Asio [38]	R, CS	HA/ M	762	18 +	ADR	56	8.6, NA	WHO
Brvar [39]	R, C	HA/E, M	520	18 +	ADR	30	5.8, NA	WHO
Cabré [40]	R, CS	HA/A	3292	70 +	ADR	197	5.9, 5.2–6.8	WHO
Cahir [13]	R, CS	HA/E	3760	65 +	ADR	377	9.6, NA	NR
Dechanont [41]	P, C	HA/M	1776	60 +	DRP	144	3.2, NA	PCNE
Fattinger [42]	R, C	HA, DH/M	4331	–	ADR	134	3.3, NA	Not clear
Gebremariam [8]	P, CS	HA/E	406	18 +	DRP	124	30.5, 27.7–36.4	Own definition
Giardina [43]	P, CS	HA, DH/M	4802	A	ADR	296	6.2, 5.5–6.8	Not clear
Hopf [44]	P, P	HA/E	1101	16 +	ADR	30	2.7, 1.8–3.7	WHO
Jolivot [45]	P, CC	HA/I	743	18 +	ADE	173	23.3, NA	WHO
Karuppannan [46]	P, CS	HA/M	1124	ALL	ADE	443	39, NA	WHO
Komagamine [47]	R, CS	HA/M	5707	18 +	ADR	287	5.0, 4.5–5.6	EA
Komagamine and Kobayashi [48]	R, CS	HA/M	1545	O	ADR	153	9.9, 8.4–11.4	EA
Kongkaew [49]	P, C	HA/M	3904	16 +	ADE	439	11.2, NA	Not clear
Lagnaoui [50]	P, C	HA, DH/M	444	15 +	ADR	116	7, NA	Own definition
Laroche [51]	P, CS	HA/M	3648	ALL	ADR	309	8.5,7.6–9.4	Own definition
Lavan [52]	P, CS	HA/O	350	16 +	ADR	75	21.5, NA	EA
Lea [53]	R, CS	HA/M	404	18 +	DRP	155	38, NA	PCNE
Leendertse [54]	P, CC	HA	29,852	18 +	DRP	714	5.6, NA	WHO for adverse event
Li [14]	R, CS	HA/E	5521	18 +	ADR	496	9, NA	NR
Luttikhuis [55]	R, C	HA/E	240	70 +	DRP	73	30.4, NA	Own definition
Marcum[56]	R, C	HA/ALL	678	65 +	DRP	40	5.9, NA	Own definition
Marcum [57]	R, C	HA/-g	678	65 +	ADR	68	10, NA	NR
Marikova [58]	R, CS	HA/M	366	78 +	ADE	43	11.7, NA	Nebeker
McLachlan [59]	P, CS	HA/M	336	16 +	ADE	96	28.6, NA	Nebeker
Menéndez-Conde [60]	P, CS	HA/E	252	18 +	ADR	49	19.4, NA	NR
Mjörndal [61]	P, CS	HA/M	681	18 +	ADR	82	12, NA	WHO
Mouton [62]	P, CS	HA/M	1951	A	ADR	162	8.4, 7.2–9.7	AF for ADR
Ocovska [63]	R, CS	HA/E	1252	A	ADR	195	15.6, 13.6–17.6	Own definition
Olivier [64]	P, CC	HA/E, M	671	15 +	ADR	41	6.1 per 100 admissions (4.4 − 8.3)	WHO
Olivier [65]	P, CS	HA/E	789	65 +	ADR	66	8.4 per 100 admissions (6.5–10.5)	EA
Onder [66]	R, CS	HA/M	28,411	O	ADR	964	3.4, NA	WHO
Pedrós [67]	P, CS	HA/-	4403	A	ADR	186	4.2, 3.7–4.8	EU
Pedrós [68]	R, CS	HA/-	60,263	65 +	ADR	1,976	3.3, 3.1–3.4	EU
Pirmohamed [69]	P, C	HA/E, M, S	18,820	13 +	ADR	1,225	6.5, 6.2–6.9	EA
Pourseyed [70]	P, CS	HA,DH/M	400	–	ADR	7	1.8, NA	WHO
Pouyanne [71]	P, CS	HA/M	3137	A	ADR	100	3.2, 2.4–4.01	NR
Santamaria-Pablos [72]	P, CS	HA/E	163	–	DRP	53	32.5, 25.8–40.0	Own definition
Singh [73]	P, CS	HA/M	3560	–	DRP	118	3.3, NA	WHO
Smeaton [74]	P, P	HA/M	100	45–64	ADR	14	14, NA	NR
Somers [75]	Survey, CS	HA/E, M	110	65 +	DRP	23	20.9, NA	WHO
van der Hooft [76]	R, C	HA/M	3515	A	ADR	115	5.1, 4.3–6.1	Own definition
Varallo [77]	P, CS	HA/M	248	18 +	ADE	115	46.4, NA	NR
Von Euler [78]	R, C	HA/E, M	168	18 +	ADR	18	11, NA	NR
Wawruch [79]	R, CS	HA/M	600	65 +	ADR	47	7.8, NA	Not clear

Adults (A); Adverse drug event (ADE); Adverse drug reaction (ADR); Aronson and Ferner for ADR (AF for ADR); Case–control study (CC); Cohort study (C); Cross-sectional study (CS); Drug-related problem (DRP); During hospitalisation (DH); Edwards and Aronson definition (EA); Emergency (E); European Union (EU); Hospital admission (HA); Intensive care (I); Jonathan R. Nebeker definition (Nebeker); Medical (M); Medication-related admission (MRA); Not reported (NR); Older adults (O); Pilot study (Pi); Prospective (P); Retrospective (R); Surgical (S); The Pharmaceutical Care Network Europe (PCNE); World Health Organization (WHO).

### Participant characteristics

Most studies recruited an adult population. Seventeen studies recruited adults of any age, 29 studies recruited specific age categories: > 18 years (n = 13), > 65 years (n = 12), > 60 years (n = 1), 45–64 years (n = 1). Two studies compared participants aged ≥ 65 versus < 65 years. The age range was not clearly reported in four studies. Two studies recruited participants younger than 15 years as adults and age 15 in two studies. These four studies were included in the review because the majority of participants were ≥ 16 years (an inclusion criterion for this review), (Supplementary material, Expanded characteristics).

### Exposure (age) characteristics

Age was variably described as a continuous variable (n = 28), a categorical variable (n = 12) and both (n = 10). The reported statistical approach to explore the association between age and study group (medication-related admission or not) was also variable, mostly bivariate analysis categorising participants as younger and older (n = 45) and the minority (n = 22) reporting multivariate analysis, (Supplementary material, Table 1).

### Outcome characteristics

Most (n = 35) studies defined medication-related problem as an ADR, as per the WHO definition, nine as a drug-related problem, and six as an ADE, (Supplementary material, Expanded characteristics). None of the included studies defined medication-related problem as medication non-adherence.

### Study quality

Most studies performed well across key domains, including having representative samples, clearly defined inclusion criteria, appropriate outcome assessment, and suitable statistical analyses. However, a small number of studies did not clearly define their inclusion criteria or specify the participant age groups. Some studies did not adequately report how confounding factors were identified or addressed, and the analysis of confounding factors was not always clearly described. In several studies, it was unclear how the sample size was calculated or whether it was adequate. Overall, the majority of studies were assessed as good quality, (Supplementary material, Table 2).

**Table 2:** The reported association between study group (MRA or non-MRA) and age

	Bivariate analysis				Multivariate regression	
Author (year)	Test	*P*-value	Crude odds ratio (95% Cl)	*P*-value	Adjusted odds ratio (95% CI)	*P*-value
Adedapo [33]	NR	NR	Aged < 64 (Reference) Aged ≥ 65 1.34 (0.73–2.5)	NSS	Aged < 64 (Reference) Aged ≥ 65 1.33 (0.5–3.52)	NSS
Ahern [34]	Student- t test	< 0.05	NR	NR	NR	NR
Alexopoulou [35]	Student- t test	< 0.05	NR	NR	NR	NSS
Alvarez [36]	Student *t* test	NSS	NR	NR	NR	NR
Angamo [37]	Mann–Whitney tests	NSS	NR	NR	NR	NR
Asio [38]	NR	NR	Mean age 1.01 (0.99–1.03)**	NSS	Mean age 1.00 (0.98–1.03)**	NSS
Brvar [39]	Pearson's correlation	NR	NR	NR	NR	< 0.05
Cabré [40]	For mean age Student- t test For > 85 years Chi-square test or Fisher’s exact test	NSS For > 85 years < 0.05	1. Mean age: 1 (0.98–1.03) 2.Aged > 85 years: 0.98 (0.74–1.31)	NSS	Not reported	NSS
Cahir [13]	NR	NR	1. Mean age: 0.97 (0.95, 0.99)** 2. Aged > 85 years: 0.74 (0.55, 1.00)	1. < 0.05 2. NS	1. Mean age: 0.96 (0.94, 0.98)** 2.Aged > 85 years: NA	1. < 0.05
Dechanont [41]	Chi‐square test	< 0.05	NR	NR	Age 60–69 Y: (Reference) 70–79 Y: 1.92 (1.06–3.47) ≥ 80 Y: 0.68 (0.25–1.86)	< 0.05
Fattinger [42]	Mann–Whitney U test	NSS	NR	NR	NR	NR
Gebremariam [8]	NR	NR	1.02 (1.01–1.03)**	< 0.05	0.99 (0.98–1.02)**	NSS
Giardina [43]	Mann–Whitney U test	NSS	Aged ≥ 85 years: 1.08 (0.83–1.42)	NSS	Aged ≥ 85 years: 0.95 (0.72–1.25)	NSS
Hopf [44]	Mann–Whitney U test	NSS	NR	NR	NR	NR
Jolivot [45]	Mann–Whitney U test	NSS	NR	NR	NR	NR
Karuppannan [46]	Chi‐square test	< 0.05	NR	NR	NR	NR
Komagamine [47]	NR	NR	Aged ≥ 65 years: 3.20 (2.18–4.69)	< 0.05	Aged ≥ 65 years: 2.00 (1.34–3.00)	< 0.05
Komagamine and Kobayashi [48]	NR	NR	Aged ≥ 65 years: 1.48 (0.95–2.30)	NSS	Aged ≥ 65 years: 0.96 (0.60 to 1.54)	NSS
Kongkaew [49]	Chi^2^ test	< 0.05	NR	NR	16–44Y: (Reference) 45–64Y: 0.44 (0.32–0.62) 65–74Y: 0.47 (0.33–0.67) 75–84Y: 0.64 (0.46–0.89) ≥ 85Yr: 0.42 (0.29–0.62)	< 0.05
Lagnaoui [50]	Kruskal–Wallis comparison	NSS	NR	NR	NR	NR
Laroche [51]	Mann–Whitney U and Kruskal- Wallis tests	NSS	NR	NR	NR	NR
Lavan [52]	Mann–Whitney test	NSS	NR	NR	NR	NR
Lea [53]	NR	NR	NR	NR	NR	NR
Leendertse [54]	Mann–Whitney U test,	< 0.01	NR	NR	Median age: 1.04 (1.03–1.05)**	< 0.05
Li [14]	Chi‐square test and tests of variance analysis,	< 0.01	NR	NR	NR	NR
Luttikhuis [55]	NR	NR	Median age: 0.98 (0.94–1.03)**	NSS	Median age: 0.96 (0.92–1.01)**	NSS
Marcum [56]	NR	NR	Aged ≥ 85 Y: 1.0 (Reference) 65–74Y: 0.91 (0.29–2.84) 75–84Y: 0.84 (0.27–2.60)	NSS	NR	NR
Marcum [57]	Chi‐square test	NSS	NR	NR	1. Aged ≥ 85 Y: 1.0 (Reference) 2. 65–74Y: 0.76 (0.33–1.74) 3. 75–84 Y: 0.62 (0.27–1.41)	2. 65–74 Y: NSS 3. 75–84 Y: NSS
Marikova [58]	Mann–Whitney U test,	NSS	NR	NR	NR	NR
McLachlan [59]	Student t-test	P = NSS	NR	NR	NR	NR
Menéndez-Conde [60]	Chi-square test	NSS	NR	NR	NR	NR
Mjörndal [61]	Wilcoxon sum	NSS	NR	NR	NR	NR
Mouton [62]	NR	NR	Median age: 1.04 (0.95–1.13)**	NSS	Median age: 1.02 (0.91–1.14)**	NSS
Ocovska [63]	NR	NR	NR	NR	NR	NR
Olivier [64]	NR	NSS	NR	NR	NR	NR
Olivier [65]	Student *t* test, Chi-square	NSS	NR	NR	NR	NR
Onder [66]	Chi-square analysis	NSS	NR	NR	1. < 65: Reference 65-79Y: 1.05 (0.90–1.23) > 80 Y: 0.91 (0.76–1.09) 2. (< 65Y vs 65–79Y: 0.98 (0.81–1.20); < 65Y vs > 80Y: 0.83 (0.67–1.04)	NSS
Pedrós [67]	Mann–Whitney and Chi2 test	< 0.05	NR	NR	< 65 years: Reference ≥ 65 years: 1.59(1.10–2.29)	< 0.05
Pedrós [68]	NR	NR	NR	NR	NR	NR
Pirmohamed [69]	Mann–Whitney U test	< 0.05	NR	NR	NR	NR
Pourseyed [70]	Student- t test	< 0.05	NR	NR	NR	NR
Pouyanne [71]	Poisson distribution	< 0.05	NR	NR	NR	NR
Santamaria-Pablos [72]	Student- t test	< 0.05	NR	NR	0.981 (NA)**	NSS
Singh [73]	NR	NSS	NR	NR	NR	NSS
Smeaton [74]	NR	NR	0.92 (0.82–1.04)**	NSS	0.96 (0.87–1.07)**	NSS
Somers [75]	NR	< 0.05	NR	NR	NR	NR
van der Hooft [76]	NR	NR	NR	NR	NR	NR
Varallo [77]	Chi-square analysis	NSS	Not older: 1.00 (0.8–2.1) Older: 1.30 (NR)	NSS	Not older: 1.00 (0.66–1.93) Older: 1.13 (NR)	NSS
Von Euler [78]	NR	NR	NR	NR	NR	NR
Wawruch [79]	Chi-square test	NSS	NR	NR	NR	NR

Abbreviation: Charlson comorbidity index (CCI); Glomerular filtration rate; (GFR); Medication-related admission; (MRA); Mini nutritional assessment; (MNA); Modification of diet in renal disease (MDRD); Not reported (NR); Not statistically significant (NSS); Potentially inappropriate medication (PIM). Increasing MRA associated with increasing age signed as ( +); increasing MRA associated with decreasing age signed as (-). * Comorbidity index: Selim physical comorbidity index, Selim psychiatric comorbidity index; Clinical visits: geriatric evaluation and management clinic visit in previous year, number of primary care visits in previous year, emergency department visits in previous year, hospitalisations in previous year, year of hospital admission. ** age was considered as a continuous variable. ^&^ < 0.05 with Adverse drug event (ADE) admissions excluding recreational drugs and overdoses

### Study findings

In total 210,514 participants were recruited across the 50 included studies, of whom 9,723 (4.62%, 95% CI 4.53, 4.71) were reported to experience medication-related admission.

### The association between age and medication-related admission

#### Bivariate analysis

Twenty-seven of the 45 studies using bivariate analysis, reported no statistically significant association between age and study group, 17 reported a statistically significant association and one study did not report the finding (Table 2).

#### Multivariate analysis

Of the 22 studies that explored the relationship between age and study group using multivariate regression, seven reported a statistically significant relationship after adjusting for potential independent variables [13, 14, 39, 41, 47, 49, 67], with a greater likelihood of medication-related admission associated with increasing age in five studies, [14, 39, 41, 47, 67] and with younger age in two studies [13, 49] (Supplementary material, Table 3). Kongkaew et al., reported a greater likelihood of medication-related admission with increased age between 16 and 84 years, which decreased for patients aged 85 years and older [49]. This study found that preventable ADR-related hospitalisations were more likely in patients aged 75–84 years compared with 16–44 year olds. Non-preventable ADR-related hospitalisations were reported as more likely in younger (16–44 years) than middle- aged (45–64 years)or older (≥ 85 years) adults [49]. Cahir et al., found that younger age was associated with a reduced likelihood of ADR-related hospital admission than other cause [13], Table 2.

The remaining 15 studies reported no statistically significant association between study group and age [8, 33, 35, 38, 40, 43, 48, 55, 57, 62, 66, 72–74, 77]. Of note, Onder et al., found that age alone cannot be considered a risk factor for ADR-related hospitalisation across all adults. However, patients who experienced severe ADR-related hospitalisations (defined as a serious clinical risk to a patient including leading to their death) were found to be statistically significantly older than those who did not (73.1 ± 12.9 vs. 69.2 ± 15.5; *p* < 0.05). In this study, age was identified as an independent risk factor for patients aged 65–79 (OR 1.50, 95% CI 1.01–2.23) and those aged 80 and older (OR 1.53, 95% CI 1.00–2.33) [66].

### Data synthesis and meta-analysis

**Fig. 2:**
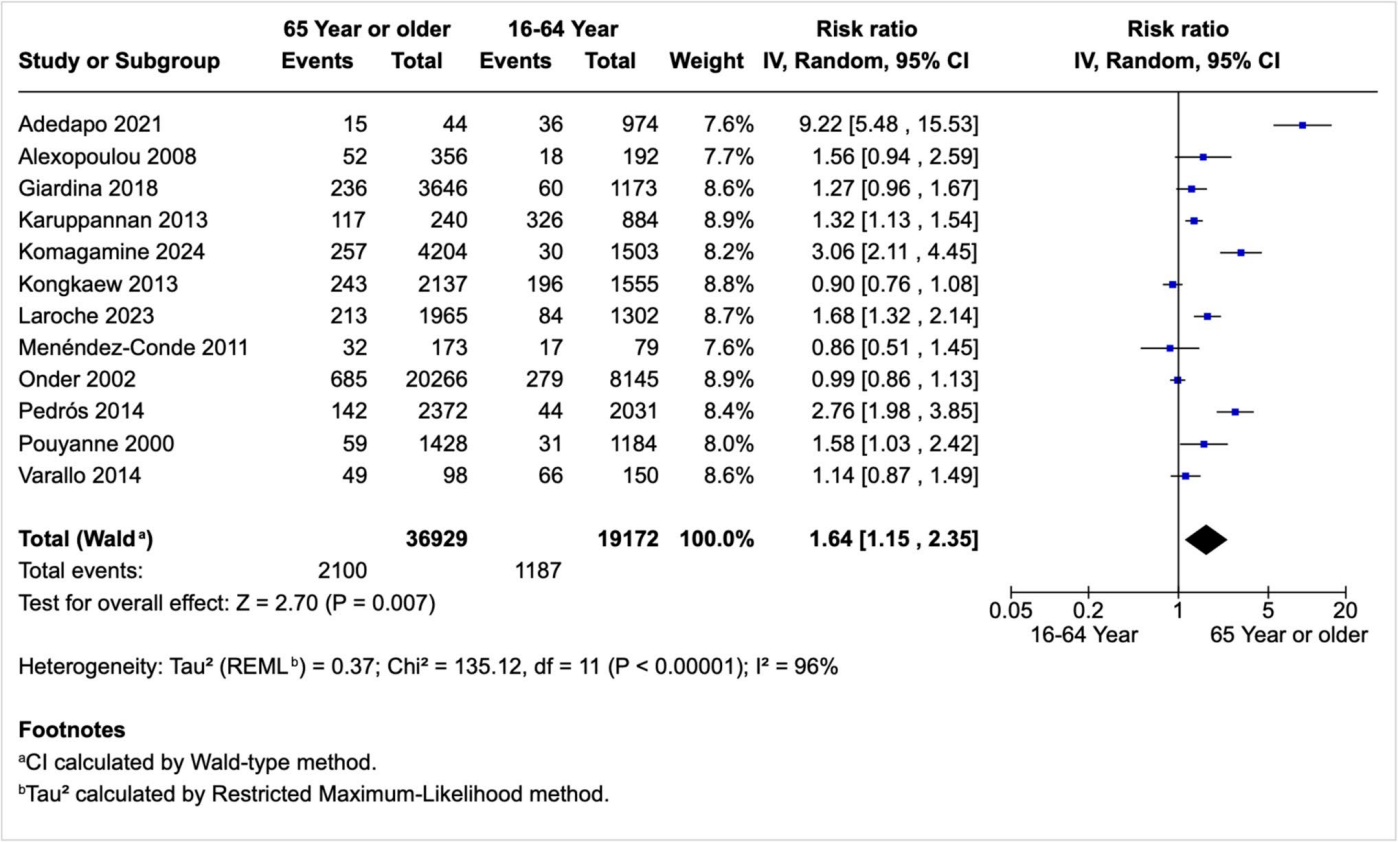
Forest plot describing the risk ratio of medication-related hospital admission by age category

Twelve studies were meta-analysed to compare, within each study, the likelihood of a younger (16–64 years) adult versus an older (≥ 65 years) adult experiencing medication-related admission relative to another cause of admission (Fig. 2). The chosen age bands were based on the predominance of age 65 years being applied to categorise age across the included studies (Table 2). The pooled RR (1.64, 95% CI 1.15–2.35), K = 12, I^2^ = 96%, n = 56,101) suggested that older adults have a higher risk of experiencing medication-related admission, relative to another reason for admission, than younger adults (Fig. 2). Heterogeneity between studies was high (τ^2^ 0.3734 (SE = 0.1724, I^2^ 95.73%, H^2^ 23.44, Q (df = 11) = 135.1173, *p*-val < 0.0001). However, the 95% prediction interval (− 0.75–1.75 on the log scale) indicated substantial heterogeneity, suggesting that future studies may generate a different finding. Meta-regression analysis identified geographic region as a significant source of heterogeneity. Compared with the reference category (Africa), studies conducted in Asia and Europe demonstrated significantly lower effect estimates (Asia: β = − 1.45, *p* = 0.002; Europe: β = − 1.78, *p* < 0.001). There was also a trend toward lower effect estimates in studies from America, although this did not reach statistical significance (β = − 1.32, *p* = 0.081). No significant associations were observed between effect size and risk of bias, outcome definition, study design, or year of publication. Visual inspection of the funnel plot asymmetry supported the finding of significant between study variation, rather than publication bias (Supplemental material, Funnel plot 1).

Sensitivity analysis, omitting Adedapo et al., 2021 and Alexopoulou et al., 2008, generated a consistent finding (RR 1.41, (95% CI 1.08–1.83), K = 10, I^2^ = 92%, n = 54,535), with similarly high heterogeneity and a wide prediction interval (0.62 -3.19), again suggesting that the finding in future studies could be different.

**Fig. 3:**
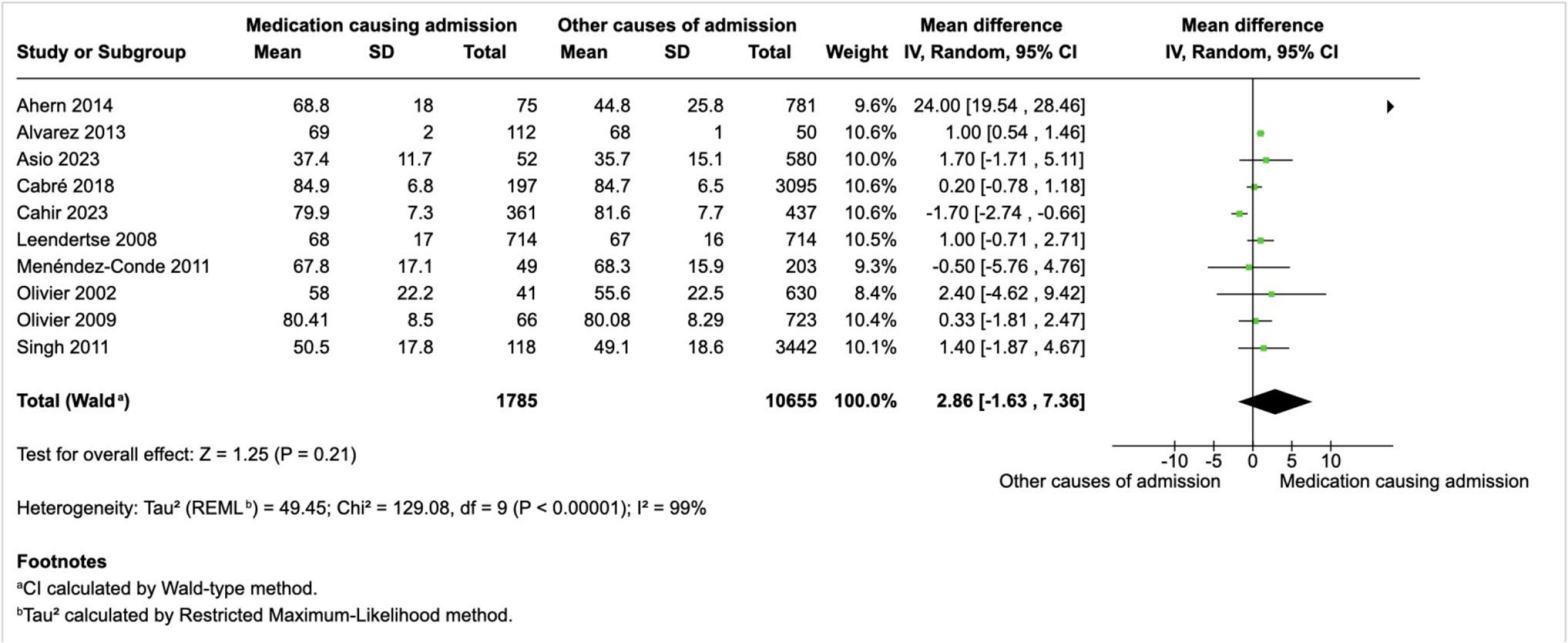
Forest plot describing the mean differences in age between the study groups

Ten studies were meta-analysed to compare the mean age between the study groups (MRA and non-MRA), (Fig. 3). The pooled mean difference (2.86 years, 95% CI − 1.63–7.36; *p* = 0.21, K = 10, I^2^ = 93%, n = 12,440) indicated no statistically significant difference between groups. There was substantial between-study heterogeneity (τ^2^ = 49.45; Q = 129.08, p < 0.001). The 95% prediction interval (− 11.63 to 17.36 years) suggested that the true effect in a future study could vary widely and include both younger and older ages in either group.

Meta-regression analyses suggested that risk of bias judgement was significantly associated with effect size (QM = 4.60, *p* = 0.032), explaining approximately 32% of the between-study heterogeneity. Studies classified as having moderate risk of bias showed larger mean differences compared with the reference category (high risk). No significant associations were observed between effect size and geographic region, outcome definition, year of publication, study design or participant age subgroup, and these variables did not explain heterogeneity (R^2^ = 0%). The asymmetry of the funnel plot was consistent with the substantial heterogeneity, with a single outlier, although it may also suggest publication bias, with smaller or negative studies being under-represented (Supplemental material, Funnel plot 2).

According to the GRADE assessment, the certainty of evidence for both outcomes was rated as very low, (Supplemental material, Table 4).

## Discussion

This systematic review and meta-analysis, comprising 50 studies and 210,514 participants, found that the majority of bivariate analyses show no association between age and medication related admission, some suggest higher risk in older adults and a minority in younger adults. Results from meta-analysis were inconsistent and overall certainty of evidence was very low. Taken together, these findings suggest that age alone is an unreliable predictor of medication-related admission without accounting for confounding and context.

Current evidence suggests that risk factors other than age are associated with medication-related admission such as polypharmacy (> 5 drugs); dementia or impaired cognition; use of antiepileptics; anticoagulants; concurrent use of NSAIDs and oral anticoagulants; insulin therapy; inadequate patient knowledge or understanding of therapy goals; medications with a narrow therapeutic window; and non-adherence [57, 80, 81]. Attempts at meta-regression and sub-group analysis in the current review were limited to study characteristics, rather than participant characteristics such as those listed above, due to a lack of reported data in the included studies. Future studies should, at a minimum, report the prevalence of polypharmacy or other measures of medication burden and complexity to advance an evidence-based approach to identify and manage medication-related admission in clinical practice. In the current review, several included studies reported a statistically significant between medication-related admission and age that was based on bivariate analysis. Bivariate analysis, while useful for exploring simple relationships between two variables, does not account for the multifactorial nature and potential confounders of medication-related admission [82]. Future studies exploring the relationship between medication-related admission and age should employ multivariate analysis to adjust for potential confounders.[83, 84].

One meta-analysis in this review found that older age is associated with a higher relative risk of medication-related admission compared to other causes while the other identified no difference in mean age between the groups. The sub-group analysis findings provide insight to help explain this apparent contradiction. Geographic region was identified as a variable contributing to heterogeneity in effect size of risk ratio for older compared to younger adults experiencing medication-related admission. Geographic variation was also evident in a global review of preventable medication related harm in health care [27], a meta-analysis of the incidence of ADRs in older adults prevalence [17] and a study of adverse-drug reaction related hospitalization [15]. Consistent with previous studies reporting a higher risk of medication-related harm, including hospitalization, for older adults or those receiving geriatric care in Africa compared to other regions, this systematic review suggested a higher risk of medication-related admission, compared to other cause, for studies undertaken in Africa than other regions [15]. Angamo and colleagues hypothesized such differences between income regions may be due varying prevalence of HIV/AIDS. Whilst this is clinically plausible due to the high risk of ADRs and drug-drug interactions with antiretroviral drugs, this theory remains to be proven in empiric studies. Consistent with the WHO’s recommendation to better understand medication-related harm across low- and middle-income countries, the findings of this review suggest the need for further research on geographic variation in the association between age and medication-related admission, particularly in low-resource settings [85]. They also support the implication for future research that context and confounding matter.

Sub-group analysis of the difference in mean age between study groups identified risk of bias judgement as a significant moderator of heterogeneity, whereby studies of moderate quality reported larger mean age differences compared with the high-risk reference group. This suggests that study quality may influence the magnitude and potentially the direction of the association and reinforces the implication for future research that methodological rigor in studies examining medication-related hospital admission is important and the points in the section below may guide researchers.

### Strengths and limitations of the included studies

Age was variably described across the included studies which limited opportunity for meta-analysis. Studies applied different age cut-offs (e.g., 60, 65, or 70 years) to define “older”, and “younger” adults. In some studies, adult was defined as ≥ 13 years of age. There was a lack of consistent age reporting identified in several studies, such as absence of reported mean ages for the comparator group or reporting only odds ratios and not mean values for age categories, which hindered study inclusion in the meta-analysis. Future studies should use standardised age categories and standardised reporting of aggregate age for both study groups. Multiple definitions of medication-related problem were used to identify medication-related admission. This is potentially problematic because such varied definitions of medication-related problems have been shown to significantly influence the interpretation and comparison of findings in previous studies [2, 86, 87]. However, the sub-group analysis in this review did not identify this as a moderator of heterogeneity in either meta-analysis. Medication-related problems are multifaceted, encompassing issues such as drug interactions, ADRs, medication overdose, and recreational drug use. A consistent global definition and taxonomy for medication-related problems may better support future understanding of the relationship between age and medication-related hospital admission [2, 76].

### Strengths and limitations of this systematic review

This review has several potential limitations and strengths. First, it excluded studies focusing on specific drugs or diseases, which may limit the generalisability of findings and understanding of context whilst supporting a focus on person-centred, holistic healthcare applicable to general prescribing practices. This latter point is pertinent because evidence demonstrates that higher medication burden is associated with medication-related harm risk, and that multimorbidity management in the form of multiple single morbidities, rather than holistic care, is associated with higher medication burden [88, 89]. Second, the search was limited to studies published in the English language and up to March 2025, so it is possible that relevant studies may have been missed. This review employed a thorough search strategy across eight databases and included citation tracking, ensuring a comprehensive study collection. Third, the heterogeneity in study populations, settings, and analytical approaches reduced the comparability of results and limited the number of meta-analysed studies. However, advanced statistical techniques, including random-effects models, addressed this heterogeneity and provided a robust analysis. The review was also potentially limited by the inclusion of studies recruiting participants as young as 13-years-old, despite reporting the recruitment of adults. This affected two studies included in the meta-analysis of the risk ratio of older versus younger adults experiencing medication-related admission compared to another cause of admission. To moderate this risk, a sensitivity analysis omitting these two studies was undertaken, and generated a consistent finding as the base analysis. Finally, although we chose the JBI Tool for prevalence studies to assess the risk of bias, several other tools were potentially applicable, for example, the Risk of Bias in Non-Randomised Studies–Exposure (ROBINS-E) [90] or the Newcastle-Ottowa Scale (NOS) [91]. It is possible that a different risk of bias judgement may have been generated from these tools. The ROBINS-E may have provided greater insight about the causal inference, i.e., the effect of age on risk of medication-related admission. The NOS and ROBINS-E would have produced a summary score, rather than the JBI tools reliance on reviewer judgement. However, we do not believe the application of another tool would have changed the overall finding that the risk of bias in the included studies was generally good.

### Implications for practice and research

The evidence in this review suggests that age alone is an unreliable predictor for medication-related admission. Contemporary research about medication-related admission over-represents older adults [58, 92]. For instance, The OPERAM project aimed to optimize pharmacological and non-pharmacological treatments for older patients focusing on reducing preventable hospital admissions [18]. ATHARM10, a validated tool, is developed to identify medication-related admission, with a specific focus on older patients [93]. Whilst it remains important to reduce medication-related hospital admissions in older adults, the uncertainty surrounding the association between age and medication-related admission, suggests that a more comprehensive, risk-based approach that considers additional patient-level factors could better support healthy-aging in the context of managing medication-related admission across the adult age span. The observed variation across geographical settings highlights the need for context-specific strategies. Despite the inclusion of fifty studies in this review, meta-analyses were limited to one-fifth of these due to the variable reporting of participant age characteristics. Better reporting of age data is needed in future studies.

## Conclusion

This review suggests that age alone may be insufficient to explain medication-related admission and supports a shift towards more comprehensive, risk-based approaches to explore and explain this issue. It encourages further research across all adult age groups to enhance the prevention, identification and management of medication-related admission to enhance care and clinical outcomes across the adult age spectrum.

## Supplementary Information

Below is the link to the electronic supplementary material.

Supplementary file1 (DOCX 319 KB)

## Data Availability

No datasets were generated or analysed during the current study.
