# Supplementary material for: The association between age and medication-related hospital admission in adults: a systematic review and meta-analysis
Source: Int J Clin Pharm. 2026 Jun 4;48(4):1267–83. doi: 10.1007/s11096-026-02167-3 (PMC13369605; doi:10.1007/s11096-026-02167-3)
Supplement: Supplementary file 1 — Supplementary file1 (DOCX 319 KB) [file 11096_2026_2167_MOESM1_ESM.docx]

###### **Supplementary material**

## Search strings used in systematic review:

**Concept 1 keywords:**

hospitalization OR hospitalisation OR hospital OR "secondary care" OR "hospital-admission" OR "hospital admission" OR admit OR "emergency admission" OR "acute care admission"

**Concept 2 keywords:**

"medication* error" OR "drug use error" OR "medication* related" OR "medicine* related" OR "drug related" OR "drug-induced problem" OR "medicine* related morbidity" OR "drug related morbidity" OR "drug related side effect" OR "drug-related side effect" OR "adverse reaction" OR "side effect of drug" OR "adverse-drug reaction" OR "drug toxicity" OR "adverse drug event" OR "adverse effect" OR "medication* adherence" OR "medication* non adherence" OR "medication* noncompliance"

**Concept 3 keywords:**

incidence OR prevalence

***Observational Designs Filter***

Cohort OR compared OR groups OR "case control" OR multivariate

**Embase:**

1. 'hospitalization'/exp
2. (hospitalization OR hospitalisation OR hospital OR 'secondary care' OR 'hospital-admission' OR 'hospital admission' OR admit OR 'emergency admission' OR 'acute care admission'):ab,ti
3. #1 OR #2
4. 'medication related problem'/exp
5. 'drug related problem'/exp
6. 'adverse drug reaction'/exp
7. 'medication error'/exp
8. 'medication compliance'/exp
9. ('medication* error' OR 'drug use error' OR 'medication* related' OR 'medicine* related' OR 'drug related' OR 'drug-induced problem' OR 'medicine* related morbidity' OR 'drug related morbidity' OR 'drug related side effect' OR 'drug-related side effect' OR 'adverse reaction' OR 'side effect of drug' OR 'adverse-drug reaction' OR 'drug toxicity' OR 'adverse drug event' OR 'adverse effect' OR 'medication* adherence' OR 'medication* non adherence' OR 'medication* noncompliance'):ab,ti
10. #4 OR #5 OR #6 OR #7 OR #8 OR #9
11. 'prevalence'/exp
12. (incidence OR prevalence):ab,ti
13. #11 OR #12
14. 'clinical article'/exp
15. 'controlled study'/exp
16. 'major clinical study'/exp
17. 'prospective study'/exp
18. 'cohort analysis'/exp
19. 'retrospective study'/exp
20. (cohort OR compared OR groups OR 'case control' OR multivariate):ab,ti
21. #14 OR #15 OR #16 OR #17 OR #18 OR #19 OR #20
22. #3 AND #10 AND #13 AND #21

Results= 6818 with language (English) and year limitations

Medline (Ovid):

1. exp HOSPITALIZATION/
2. (hospitalization or hospitalisation or hospital or "secondary care" or "hospital-admission" or "hospital admission" or admit or "emergency admission" or "acute care admission")ab,ti.
3. #1 OR #2
4. exp Medication Errors/
5. exp "Drug-Related Side Effects and Adverse Reactions"/
6. exp Medication Adherence/
7. ("medication* error" or "drug use error" or "medication* related" or "medicine* related" or "drug related" or "drug-induced problem" or "medicine* related morbidity" or "drug related morbidity" or "drug related side effect" or "drug-related side effect" or "adverse reaction" or "side effect of drug" or "adverse-drug reaction" or "drug toxicity" or "adverse drug event" or "adverse effect" or "medication* adherence" or "medication* non adherence" or "medication* noncompliance")ab,ti.
8. #4 OR #5 OR #6 OR #7
9. exp PREVALENCE/
10. incidence OR prevalence
11. #9 OR #10
12. exp Cohort Studies/
13. exp Case-Control Studies/
14. exp Comparative Study/
15. exp Prospective Studies/
16. exp retrospective Studies/
17. exp Risk Factors/
18. (cohort or compared or groups or "case control" or multivariate)ab,ti.
19. #12 OR #13 OR #14 OR #15 OR #16 OR #17 OR #18
20. #3 AND #8 AND #11 AND #19

|  |
| --- |

Results= 1758 with language and year limitations

----------------------------------------------------------------------------------------------------------------

Web of Science:

|  | 1. TS= (hospitalization OR hospitalisation OR hospital OR "secondary care" OR "hospital-admission" OR "hospital admission" OR admit OR "emergency admission" OR "acute care admission") 2. TS= ("medication* error" OR "drug use error" OR "medication* related" OR "medicine* related" OR "drug related" OR "drug-induced problem" OR "medicine* related morbidity" OR "drug related morbidity" OR "drug related side effect" OR "drug-related side effect" OR "adverse reaction" OR "side effect of drug" OR "adverse-drug reaction" OR "drug toxicity" OR "adverse drug event" OR "adverse effect" OR "medication* adherence" OR "medication* non adherence" OR "medication* noncompliance") 3. TS= (incidence OR prevalence) 4. #1 AND #2 AND #3   Results= [3,472](http://apps.webofknowledge.com.elib.tcd.ie/summary.do?product=UA&doc=1&qid=5&SID=E33G1mZkWpebWAPVoGq&search_mode=CombineSearches&update_back2search_link_param=yes) with language and year limitations  ---------------------------------------------------------------------------------------------------------- |
| --- | --- |

Scopus:

1. TITLE-ABS (hospitalization OR hospitalisation OR hospital OR "secondary care" OR "hospital-admission" OR "hospital admission" OR admit OR "emergency admission" OR "acute care admission")
2. TITLE-ABS ("medication* error" OR "drug use error" OR "medication* related" OR "medicine* related" OR "drug related" OR "drug-induced problem" OR "medicine* related morbidity" OR "drug related morbidity" OR "drug related side effect" OR "drug-related side effect" OR "adverse reaction" OR "side effect of drug" OR "adverse-drug reaction" OR "drug toxicity" OR "adverse drug event" OR "adverse effect" OR "medication* adherence" OR "medication* non adherence" OR "medication* noncompliance")
3. TITLE-ABS (incidence OR prevalence)
4. #1 AND #2 AND #3

Results = [3,374](https://www-scopus-com.elib.tcd.ie/search/history/results.uri?origin=searchhistory&shid=7)  with language and year limitations

----------------------------------------------------------------------------------------------------------------

CINAHL:

1. (MH "Hospitalization+")
2. TI (hospitalization OR hospitalisation OR hospital OR "secondary care" OR "hospital-admission" OR "hospital admission" OR admit OR "emergency admission" OR "acute care admission")
3. AB (hospitalization OR hospitalisation OR hospital OR "secondary care" OR "hospital-admission" OR "hospital admission" OR admit OR "emergency admission" OR "acute care admission")
4. S1 OR S2 OR S3
5. (MH "Adverse Drug Event+")
6. (MH "Medication Errors+")
7. (MH "Medication Compliance")
8. TI ("medication* error" OR "drug use error" OR "medication* related" OR "medicine* related" OR "drug related" OR "drug-induced problem" OR "medicine* related morbidity" OR "drug related morbidity" OR "drug related side effect" OR "drug-related side effect" OR "adverse reaction" OR "side effect of drug" OR "adverse-drug reaction" OR "drug toxicity" OR "adverse drug event" OR "adverse effect" OR "medication* adherence" OR "medication* non adherence" OR "medication* noncompliance")
9. AB ("medication* error" OR "drug use error" OR "medication* related" OR "medicine* related" OR "drug related" OR "drug-induced problem" OR "medicine* related morbidity" OR "drug related morbidity" OR "drug related side effect" OR "drug-related side effect" OR "adverse reaction" OR "side effect of drug" OR "adverse-drug reaction" OR "drug toxicity" OR "adverse drug event" OR "adverse effect" OR "medication* adherence" OR "medication* non adherence" OR "medication* noncompliance")
10. S5 OR S6 OR S7 OR S8 OR S9
11. (MH "Prevalence")
12. TI (incidence OR prevalence)
13. AB (incidence OR prevalence)
14. S11 OR S12 OR S13
15. (MH "Prospective Studies+")
16. (MH "Retrospective Design")
17. (MH "Case Control Studies+")
18. TI (cohort OR compared OR groups OR "case control" OR multivariate)
19. AB (cohort OR compared OR groups OR "case control" OR multivariate)
20. S15 OR S16 OR S17 OR S18 OR S19
21. S4 AND S10 AND S14 AND S20

Results= 724 with language and year limitations

----------------------------------------------------------------------------------------------------------------

[PsycINFO](javascript:__doPostBack('ctl00$ctl00$MainContentArea$MainContentArea$SelectDbControl$dbList$ctl02$ctl00$titleLink','')):

1. DE "Hospitalization" OR DE "Commitment (Psychiatric)" OR DE "Hospital Admission" OR DE "Hospital Discharge" OR DE "Psychiatric Hospitalization"
2. TI (hospitalization OR hospitalisation OR hospital OR "secondary care" OR "hospital-admission" OR "hospital admission" OR admit OR "emergency admission" OR "acute care admission")
3. AB (hospitalization OR hospitalisation OR hospital OR "secondary care" OR "hospital-admission" OR "hospital admission" OR admit OR "emergency admission" OR "acute care admission")
4. S1 OR S2 OR S3
5. DE "Side Effects (Drug)" OR DE "Drug Addiction" OR DE "Drug Allergies" OR DE "Drug Dependency" OR DE "Drug Sensitivity"
6. DE "Treatment Compliance"
7. TI ("medication* error" OR "drug use error" OR "medication* related" OR "medicine* related" OR "drug related" OR "drug-induced problem" OR "medicine* related morbidity" OR "drug related morbidity" OR "drug related side effect" OR "drug-related side effect" OR "adverse reaction" OR "side effect of drug" OR "adverse-drug reaction" OR "drug toxicity" OR "adverse drug event" OR "adverse effect" OR "medication* adherence" OR "medication* non adherence" OR "medication* noncompliance")
8. AB ("medication* error" OR "drug use error" OR "medication* related" OR "medicine* related" OR "drug related" OR "drug-induced problem" OR "medicine* related morbidity" OR "drug related morbidity" OR "drug related side effect" OR "drug-related side effect" OR "adverse reaction" OR "side effect of drug" OR "adverse-drug reaction" OR "drug toxicity" OR "adverse drug event" OR "adverse effect" OR "medication* adherence" OR "medication* non adherence" OR "medication* noncompliance")
9. S5 OR S6 OR S7 OR S8
10. TI (incidence OR prevalence)
11. AB (incidence OR prevalence)
12. S10 OR S11
13. DE "Prospective Studies" OR DE "Retrospective Studies"
14. TI (cohort OR compared OR groups OR "case control" OR multivariate)
15. AB (cohort OR compared OR groups OR "case control" OR multivariate)
16. S13 OR S14 OR S15
17. S4 AND S9 AND S12 AND S16

Results= 236 with language and year limitations.

----------------------------------------------------------------------------------------------------------------

Cochrane:

1. [mh “hospitalization]
2. (hospitalization OR hospitalisation OR hospital OR "secondary care" OR "hospital-admission" OR "hospital admission" OR admit OR "emergency admission" OR "acute care admission"):ti,ab,kw
3. #1 OR #2
4. [mh “medication errors]
5. [mh “Drug-Related Side Effects and Adverse Reactions]
6. [mh “medication adherence]
7. ("medication* error" OR "drug use error" OR "medication* related" OR "medicine* related" OR "drug related" OR "drug-induced problem" OR "medicine* related morbidity" OR "drug related morbidity" OR "drug related side effect" OR "drug-related side effect" OR "adverse reaction" OR "side effect of drug" OR "adverse-drug reaction" OR "drug toxicity" OR "adverse drug event" OR "adverse effect" OR "medication* adherence" OR "medication* non adherence" OR "medication* noncompliance"):ti,ab,kw
8. #4 OR #5 OR #6 OR #7
9. [mh “prevalence]
10. (incidence OR prevalence):ti,ab,kw
11. #9 OR #10
12. [mh “cohort studies]
13. [mh “case-control studies]
14. [mh “comparative study]
15. [mh “prospective studies]
16. [mh “retrospective studies]
17. [mh “risk factors]
18. cohort OR compared OR groups OR "case control" OR multivariate
19. #12 OR #13 OR #14 OR #15 OR #16 OR #17 OR #18
20. #3 AND #8 AND #11 AND #19

Results= 237 reviews with year limitation.

Global health

1. [title:(hospitalization OR hospitalisation OR hospital OR "secondary care" OR "hospital-admission" OR "hospital admission" OR admit OR "emergency admission" OR "acute care admission")](https://www.cabdirect.org/cabdirect/search/?q=title:(hospitalization%20OR%20hospitalisation%20OR%20hospital%20OR%20%22secondary%20care%22%20OR%20%22hospital-admission%22%20OR%20%22hospital%20admission%22%20OR%20admit%20OR%20%22emergency%20admission%22%20OR%20%22acute%20care%20admission%22)&sort=Relevance)
2. [ab:(hospitalization OR hospitalisation OR hospital OR "secondary care" OR "hospital-admission" OR "hospital admission" OR admit OR "emergency admission" OR "acute care admission")](https://www.cabdirect.org/cabdirect/search/?q=ab:(hospitalization%20OR%20hospitalisation%20OR%20hospital%20OR%20%22secondary%20care%22%20OR%20%22hospital-admission%22%20OR%20%22hospital%20admission%22%20OR%20admit%20OR%20%22emergency%20admission%22%20OR%20%22acute%20care%20admission%22)&sort=Relevance)
3. #1 OR #2
4. title:("medication error" OR "drug use error" OR "medication related" OR "medicine related" OR "drug related" OR "drug-induced problem" OR "medicine related morbidity" OR "drug related morbidity" OR "drug related side effect" OR "drug-related side effect" OR "adverse reaction" OR "side effect of drug" OR "adverse-drug reaction" OR "drug toxicity" OR "adverse drug event" OR "adverse effect" OR "medication adherence" OR "medication non adherence" OR "medication noncompliance")
5. ab:( "medication error" OR "drug use error" OR "medication related" OR "medicine related" OR "drug related" OR "drug-induced problem" OR "medicine related morbidity" OR "drug related morbidity" OR "drug related side effect" OR "drug-related side effect" OR "adverse reaction" OR "side effect of drug" OR "adverse-drug reaction" OR "drug toxicity" OR "adverse drug event" OR "adverse effect" OR "medication adherence" OR "medication non adherence" OR "medication noncompliance")
6. #4 OR #5
7. title:(incidence OR prevalence)
8. ab:( incidence OR prevalence)
9. #7 OR #8
10. #3 AND #6 AND #9

Results= 714 with language and year limitat

## Expanded study, participant and outcome characteristics

**Study characteristics**

The majority of studies were cross-sectional (n=33) [1-33], followed by cohort (n=12) [34-45], case-control (n=3) [46-48], and pilot studies (n=2) [49, 50]. Study sample size ranged from 100 to 60,263 participants (Table 1). Seventeen studies were published between 2000–2009 [49, 35, 37, 39, 43-45, 47, 48, 2, 20, 23, 24, 27-29, 33], 22 between 2010–2020 [50, 38, 41, 42, 46, 1, 3, 4, 6, 9, 10, 12, 14, 15, 18, 19, 21, 25, 26, 30-32], and 11 since 2021 [34, 36, 40, 5, 7, 8, 11, 13, 16, 17, 22]. Geographically, 30 studies (63%) were conducted in Europe [6, 2, 1, 29, 43, 39, 33, 31, 28, 26, 24, 48, 23, 20, 47, 38, 25, 46, 49, 37, 9, 45, 15, 14, 50, 22, 7, 17, 40, 13]. The remainder were undertaken in Africa (n=5) [4, 21, 34, 5, 8], South-east Asia (n=3) [30, 10, 36], Western Pacific (n=4) [18, 12, 16, 11], North America (n=3) [42, 41, 32], South America (n=1) [3] or the Eastern Mediterranean region (n=1) [27].Three studies did not report their geographical location [35, 44, 19].

**Participant characteristics**

Most studies recruited an adult population. Seventeen studies recruited adults of any age [44, 28, 27, 43, 48, 18, 14, 13, 21, 22, 39, 38, 34, 1, 2, 49, 10], 29 studies recruited specific age categories: >18 years (n=13) [4, 5, 35, 8, 46, 11, 45, 15, 47, 16, 19, 20, 32], >65 years (n=12) [6, 7, 12, 40-42, 17, 23, 24, 26, 31, 33], >60 years (n=1) [36], 45–64 years (n=1) [50] . Two studies compared participants aged ≥65 versus <65 years [25, 9], . The age range was not clearly reported in four studies [3, 37, 29, 30]. Two studies recruited participants younger than 15 years as adults [27, 34] and age 15 in two studies [39, 2]). These four studies were included in the review because the majority of participants were ≥16 years (an inclusion criterion for this review).

**Outcome characteristics**

Most (n=35) studies defined medication-related problem as an ADR, as per the WHO definition [6, 35, 4, 3, 2, 1, 44, 43, 39, 33, 28, 27, 19, 26, 24, 48, 23, 21, 20, 42, 25, 49, 37, 9, 45, 12, 14, 50, 34, 13, 16, 7, 5, 22, 11], nine as a drug-related problem [29, 41, 31, 30, 47, 15, 36, 40, 8], and six as an ADE [32, 38, 10, 46, 18, 17]. None of the included studies defined medication-related problem as medication non-adherence.

## *Supplemental table 1.* The reported association between study group and age.

|  | Bivariate analysis | | | | Multivariate regression | | |
| --- | --- | --- | --- | --- | --- | --- | --- |
| Author (year) | **Test** | **P-value** | **Crude odds**  **ratio (95% Cl)** | **P-value** | **Adjusted odds ratio (95% CI)** | **P-value** | **Independent variables assessed** |
| Adedapo, 2021 | NR | NR | Aged < 64 (Reference)  Aged ≥ 65  1.34 (0.73-2.5) | NSS | Aged < 64 (Reference)  Aged ≥ 65  1.33 (0.5-3.52) | NSS | Gender, educational level, income, alcohol intake, cigarette smoking, fixed-dose combination, duration of hospital stays, number of drugs used a month before admission, number of drugs used on admission, CCI score |
| Ahern, 2014 | Student- t test | <0.05 | NR | NR | NR | NR | NR |
| Alexopoulou, 2008 | Student- t test | <0.05 | NR | NR | NR | NSS | Number of drugs |
| Alvarez, 2013 | Student *t* test | NSS | NR | NR | NR | NR | NR |
| Angamo, 2017 | Mann-Whitney tests | NSS | NR | NR | NR | NR | NR |
| Asio et al., 2023 | NR | NR | Mean age  1.01 (0.99–1.03)** | NSS | Mean age  1.00 (0.98–1.03)** | NSS | Gender, HIV and antiretroviral therapy, antituberculosis therapy, self-medication, history of drug allergies, comorbidity score and number of drugs |
| Brvar , 2009 | Pearson's correlation was used to correlate age in continuous age variable. | NR | NR | NR | NR | <0.05 | Gender, number of drugs, number of diagnoses, renal failure, liver failure, alcohol abuse, death |
| Cabré , 2018 | For mean age Student- t test  For >85 years Chi-square test or Fisher’s exact test. | NSS  For >85 years <0.05 | 1. Mean age:  1 (0.98–1.03)  2.Aged >85 years:  0.98 (0.74–1.31) | NSS | Not reported | NSS | Gender, previous falls, number of drugs pre-admission, sedatives, antidepressants, PIM-Beer’s list, MNA < 23.5, GFR- MDRD. |
| Cahir et al., 2023 | NR | NR | 1. Mean age:  0.97 (0.95, 0.99)**  2. Aged >85 years:  0.74 (0.55, 1.00) | 1. <0.05  2. NS | 1. Mean age:  0.96 (0.94, 0.98)**  2.Aged >85 years:  NA | 1.<0.05 | Gender, functional ability (geriatric syndromes), falls history, comorbidity, polypharmacy, types of medication on admission, and self-medication use. |
| Dechanont, 2021 | Chi‐square test | <0.05 | NR | NR | Age 60–69 Y:  (Reference)  70–79 Y:  1.92 (1.06–3.47)  ≥80 Y:  0.68 (0.25–1.86) | <0.05 | Gender, family, number of prescribed medications, hospital dispensed medications, drug store dispensed medications, starting new drugs within 1 week, and starting new drugs within 1 month. |
| Fattinger, 2000 | Mann–Whitney U test | NSS | NR | NR | NR | NR | NR |
| Gebremariam et al., 2024 | NR | NR | 1.02 (1.01-1.03)** | <0.05 | 0.99 (0.98-1.02)** | NSS | Marital status, uncorrected visual impairment, uncorrected mobility impairment, renal impairment, chronic disease, history of traditional medication use, history of hospitalisation, number of medication used before admission and duration for medication used before admission |
| Giardina, 2018 | Mann–Whitney U test | NSS | Aged ≥ 85 years: 1.08 (0.83–1.42) | NSS | Aged ≥ 85 years:  0.95 (0.72–1.25) | NSS | Gender, number of drugs taken, and CCI score |
| Hopf, 2008 | Mann–Whitney U test | NSS | NR | NR | NR | NR | NR |
| Jolivot, 2016 | Mann–Whitney U test | NSS | NR | NR | NR | NR | NR |
| Karuppannan, 2013 | Chi‐square test | <0.05 | NR | NR | NR | NR | NR |
| Komagamine, 2024 | NR | NR | Aged ≥ 65 years:  3.20 (2.18-4.69) | <0.05 | Aged ≥ 65 years:  2.00 (1.34-3.00) | <0.05 | Gender, ambulance use, CCI score, chronic kidney disease and polypharmacy |
| Komagamine, 2019 | NR | NR | Aged ≥ 65 years: 1.48 (0.95 to 2.30) | NSS | Aged ≥ 65 years: 0.96 (0.60 to 1.54) | NSS | Gender, Polypharmacy |
| Kongkaew, 2013 | Chi^2^ test | <0.05 | NR | NR | 16–44Y:  (Reference)  45–64Y: 0.44 (0.32–0.62)  65–74Y: 0.47 (0.33–0.67)  75–84Y: 0.64 (0.46–0.89)  ≥ 85Yr: 0.42 (0.29–0.62) | <0.05 | Length of time since starting new drugs, number of prescription drugs, hospital site |
| Lagnaoui, 2000 | Kruskal-Wallis comparison | NSS | NR | NR | NR | NR | NR |
| Lavan , 2019 | Mann-Whitney U and Kruskal- Wallis tests | NSS | NR | NR | NR | NR | NR |
| Lea, 2019 | Mann-Whitney test | NSS | NR | NR | NR | NR | NR |
| Leendertse, 2008 | NR | NR | NR | NR | NR | NR | NR |
| Li et al., 2021 | Mann–Whitney U test, | <0.01 | NR | NR | Median age:  1.04 (1.03–1.05)** | <0.05 | Gender, number of medications |
| Laroche 2023 | Chi‐square test and tests of variance analysis, | <0.01 for age as a continuous and categorical variable | NR | NR | NR | NR | NR |
| Luttikhuis et al., 2022 | NR | NR | Median age:  0.98 (0.94–1.03)** | NSS | Median age:  0.96 (0.92–1.01)** | NSS | Gender, education, nursing home resident (or not), geriatric (functional dependency, cognitive impairment and dementia), disease severity (CCI score), previous hospital admissions, arrival by ambulance and drug-related (number of medications, polypharmacy) |
| Marcum,  2012b | NR | NR | Aged ≥ 85 Y:  1.0 (Reference)  65–74Y:  0.91 (0.29–2.84)  75–84Y:  0.84 (0.27–2.60) | NSS | NR | NR | NR |
| Marcum, 2012a | Chi‐square test | NSS | NR | NR | 1. Aged ≥ 85 Y:  1.0 (Reference)  2. 65–74Y: 0.76 (0.33–1.74)  3. 75–84 Y: 0.62 (0.27–1.41) | 2. 65–74 Y: NSS  3. 75–84 Y: NSS | Primary independent variable: Polypharmacy Control variables adjusted for sex, race, Hispanic, unmarried, comorbidity index*, non-exempt copayment status, clinical visits* |
| Marikova et al,. 2021 | Mann–Whitney U test, | NSS | NR | NR | NR | NR | NR |
| McLachlan, 2014 | Student t-test | P = NSS with all ADE-associated admissions^&^ | NR | NR | NR | NR | NR |
| Menéndez-Conde, 2011 | Chi-square test | NSS | NR | NR | NR | NR | NR |
| Mjörndal, 2002 | Wilcoxon sum | NSS | NR | NR | NR | NR | NR |
| Mouton, 2016 | NR | NR | Median age:  1.04 (0.95 – 1.13)** | NSS | Median age:  1.02 (0.91 – 1.14)** | NSS | Sex, antituberculosis therapy, antiretroviral therapy and HIV, drug count, and comorbidity score. |
| Ocovska 2022 | NR | NR | NR | NR | NR | NR | NR |
| Olivier, 2002 | NR | NSS | NR | NR | NR | NR | NR |
| Olivier, 2009 | Student *t* test, Chi-square | NSS | NR | NR | NR | NR | NR |
| Onder, 2002 | Chi-square analysis | NSS | NR | NR | 1. < 65:  Reference  65-79Y:  1.05 (0.90–1.23)  >80 Y:  0.91 (0.76–1.09)  2. (< 65Y vs 65–79Y:  0.98 (0.81–1.20);  < 65Y vs >80Y:  0.83 (0.67–1.04) | NSS | Sex, drinking and smoking habits, education, CCI score, number of drugs taken at hospital admission, and number of admissions in the last year |
| Pedrós, 2014 | Mann–Whitney and Chi2 test | <0.05 | NR | NR | <65 years: Reference  ≥65 years: 1.59(1.10-2.29) | <0.05 | Number of drugs at admission, length of in-hospital stays, patients requiring stay in special units, length of stay in special units. |
| Pedrós, 2016 | NR | NR | NR | NR | NR | NR | NR |
| Pirmohamed, 2004 | Mann-Whitney U test | <0.05 | NR | NR | NR | NR | NR |
| Pourseyed, 2009 | Student- t test | <0.05 | NR | NR | NR | NR | NR |
| Pouyanne, 2000 | Poisson distribution | <0.05 | NR | NR | NR | NR | NR |
| Santamaria-Pablos, 2009 | Student- t test | <0.05 | NR | NR | 0.981 (NA)** | NSS | Underlying disease, smoking habit, educational, level comorbidity, complex medication, phytotherapy |
| Singh, 2011 | NR | NSS | NR | NR | NR | NSS | sex, the number of drugs, educational and socioeconomic status |
| Smeaton, 2020 | NR | NR | 0.92 (0.82–1.04)** | NSS | 0.96 (0.87–1.07)** | NSS | Sex, CCI score, polypharmacy, potentially inappropriate prescribing |
| Somers, 2010 | NR | <0.05 | NR | NR | NR | NR | NR |
| van der Hooft, 2008 | NR | NR | NR | NR | NR | NR | NR |
| Varallo, 2014 | Chi-square analysis | NSS | Not older: 1.00 (0.8-2.1)  Older:  1.30 (NR) | NSS | Not older: 1.00 (0.66-1.93)  Older:  1.13 (NR) | NSS | Sex, ethanol consumption, smoking habit, the number of drugs used, and the length of stay |
| von Euler, 2006 | NR | NR | NR | NR | NR | NR | NR |
| Wawruch, 2009 | Chi-square test | NSS | NR | NR | NR | NR | NR |

## Supplemental table 2. Quality assessment

| Study ID | Was the sample representative of the target population? | Were the criteria for inclusion in the sample clearly defined? | Was the sample size adequate? | Were outcomes assessed using objective criteria? | If comparisons are being made, was there sufficient description of the groups? | Withdrawals: Outcomes Described and Included in Analyses? | Were outcomes measured in a reliable way? | Are all confounding factors or subgroups identified and addressed? | Was the data analysis conducted with sufficient coverage of the confounding factors or subgroups? | Was there appropriate statistical analysis? |
| --- | --- | --- | --- | --- | --- | --- | --- | --- | --- | --- |
| Adedapo 2020 | Yes | Yes | Unclear | Yes | Yes | Not applicable | Yes | Yes | Yes | Yes |
| Ahern 2014 | Yes | Yes | Unclear | Yes | Yes | Not applicable | Yes | Not applicable | Not applicable | Yes |
| Alexopoulou 2008 | Yes | Yes | Not applicable | Yes | Yes | Not applicable | Yes | Yes | Yes | Yes |
| Alvarez 2013 | Yes | Yes | Unclear | Yes | Yes | Not applicable | Yes | Yes | Yes | Yes |
| Angamo 2017 | Yes | Yes | Unclear | Yes | Yes | Not applicable | Yes | Yes | Yes | Yes |
| Asio 2023 | Yes | Yes | Not applicable | Yes | Yes | Not applicable | Yes | Yes | Yes | Yes |
| Brvar 2009 | Yes | Yes | Unclear | Yes | Yes | Not applicable | Yes | Yes | Yes | Yes |
| Cabré 2018 | Yes | Yes | Unclear | Yes | Yes | Not applicable | Yes | Yes | Yes | Yes |
| Cahir 2023 | Yes | Yes | Not applicable | Yes | Yes | Not applicable | Yes | Yes | Yes | Yes |
| Dechanont 2021 | Yes | Yes | Unclear | Yes | Yes | Not applicable | Yes | Yes | Yes | Yes |
| Fattinger 2000 | Yes | Yes | Unclear | Yes | Yes | Not applicable | Yes | Unclear | Unclear | Yes |
| Gebremariam 2014 | Yes | Yes | Yes | Yes | Not applicable | Not applicable | Yes | Yes | Yes | Yes |
| Giardina 2018 | Yes | Yes | Unclear | Yes | Yes | Not applicable | Unclear | Yes | Yes | Yes |
| Hopf 2008 | Yes | Yes | Not applicable | Yes | Yes | Not applicable | Yes | Not applicable | Not applicable | Yes |
| Jolivot 2016 | NO | Yes | Yes | Yes | Yes | Not applicable | Yes | Not applicable | Not applicable | Yes |
| Karuppannan 2013 | Yes | Yes | Yes | Yes | Yes | Not applicable | Yes | Not applicable | Not applicable | Yes |
| Komagamine 2024 | Yes | Yes | Yes | Yes | Yes | Not applicable | Yes | Yes | Yes | Yes |
| Komagamine 2019 | Yes | Yes | Unclear | Yes | Yes | Not applicable | Yes | Yes | Yes | Yes |
| Kongkaew 2013 | Yes | Yes | Unclear | Yes | Yes | Yes | Yes | Yes | Yes | Yes |
| Lagnaoui 2000 | Yes | Yes | Unclear | Yes | Yes | Not applicable | Unclear | Not applicable | Not applicable | Yes |
| Lavan 2019 | NO | Yes | Yes | Yes | Yes | Not applicable | Unclear | Not applicable | Not applicable | Yes |
| Lea 2019 | Yes | Yes | Unclear | Yes | Yes | Not applicable | Yes | Yes | Yes | Yes |
| Laroche 2023 | Yes | Yes | Yes | Yes | Yes | Not applicable | Yes | Not applicable | Not applicable | Yes |
| Leendertse 2008 | Yes | Yes | Unclear | Yes | Yes | Yes | Yes | Yes | Yes | Yes |
| Li 2021 | Yes | Yes | Not applicable | Yes | Yes | Not applicable | NO | Yes | Yes | Yes |
| Luttikhuis 2022 | Yes | Yes | Yes | Yes | Yes | Not applicable | Yes | Yes | Yes | Yes |
| Marcum 2012 | Unclear | Yes | Unclear | Yes | Yes | Not applicable | Yes | Yes | Yes | Yes |
| Marcum 2012 | NO | Yes | Unclear | Yes | No | Not applicable | Yes | Yes | Yes | Yes |
| McLachlan 2014 | Yes | Unclear | Unclear | Yes | Yes | Not applicable | Unclear | Not applicable | Not applicable | Yes |
| Marikova 2021 | Yes | Yes | Not applicable | Yes | Yes | Not applicable | Yes | Not applicable | Not applicable | Yes |
| Mjorndal 2002 | Yes | Yes | Unclear | Yes | Yes | Not applicable | Unclear | Not applicable | Not applicable | Yes |
| Mouton 2016 | Yes | Yes | Yes | Yes | Yes | Not applicable | Yes | Yes | Yes | Yes |
| Ocovska 2022 | Yes | Yes | Yes | Yes | Yes | Not applicable | Yes | Not applicable | Not applicable | Yes |
| Olivier 2002 | Yes | Yes | Yes | Yes | Unclear | Not applicable | Yes | Unclear | Unclear | Unclear |
| Olivier 2009 | Yes | Yes | Yes | Yes | Yes | Not applicable | Unclear | Yes | Yes | Yes |
| Onder 2002 | Yes | Unclear | Unclear | Yes | Not applicable | Not applicable | Not applicable | Yes | Yes | Yes |
| Pedrós 2016 | Yes | Unclear | Unclear | Yes | Yes | Not applicable | Not applicable | Not applicable | Not applicable | Yes |
| Pedros 2014 | Yes | Yes | Yes | Yes | Yes | Not applicable | Unclear | Yes | Yes | Yes |
| Menéndez-Conde 2011 | Yes | Yes | Unclear | Yes | Yes | Not applicable | Yes | Yes | Yes | Yes |
| Pirmohamed 2004 | Yes | Yes | Unclear | Yes | Yes | Not applicable | Yes | Not applicable | Not applicable | Yes |
| Pourseyed 2009 | Yes | Yes | Unclear | Yes | Yes | Not applicable | Not applicable | Not applicable | Not applicable | Yes |
| Pouyanne 2000 | Yes | Yes | Unclear | Yes | Yes | Not applicable | Not applicable | Not applicable | Not applicable | Yes |
| Santamaria-Pablos 2009 | Yes | Yes | Not applicable | Yes | Unclear | Not applicable | Yes | Yes | Yes | Unclear |
| Singh 2011 | Yes | Yes | Unclear | Yes | Yes | Not applicable | Not applicable | Unclear | Unclear | Yes |
| Smeaton 2020 | Yes | Yes | Not applicable | Yes | Not applicable | Not applicable | Yes | Yes | Yes | Yes |
| Somers 2010 | Yes | Unclear | Not applicable | Yes | Yes | Not applicable | Yes | Not applicable | Not applicable | Yes |
| vanderHooft 2008 | Yes | Unclear | Unclear | Yes | Not applicable | Not applicable | Yes | Not applicable | Not applicable | Yes |
| Varallo 2014 | Yes | Yes | Not applicable | Yes | Yes | Not applicable | Not applicable | Yes | Yes | Yes |
| Von Euler 2006 | Yes | Unclear | Not applicable | Yes | Yes | Not applicable | Not applicable | Not applicable | Not applicable | Unclear |
| Wawruch 2009 | Yes | Yes | Yes | Yes | Yes | Not applicable | Unclear | Yes | Yes | Yes |

##

## Supplemental table 3. Bivariate analysis applied

| Author (year) | Age variable  As Continuous | Test used | P-value | Age variable  As Categorical | Test used | P-value |
| --- | --- | --- | --- | --- | --- | --- |
| Adedapo, 2021 | NA | NA | NA | Categorical | Bivariate analyses using regression | NS |
| Ahern, 2014 | Continuous (Mean+SD) and Median 95%cl | Student *t* test, non-parametric test | Sig | NA | NA | NA |
| Alexopoulou, 2008 | Continuous Means (95% confidence interval (CI). | Student's t-test | Sig | Categorical | Chi-square analysis | NS |
| Alvarez, 2013 | Continuous (Mean ± standard error) | Student *t* test | NS | NA | NA | NA |
| Angamo, 2017 | Continuous Median (IQR) | Mann-Whitney tests | NS | NA | NA | NA |
| Asio 2023 | Continuous (Mean) and Median | NA | NA | NA | NA | NA |
| Brvar, 2009 | Continuous (Mean and range). | Pearson's correlation was used to correlate age | Non-reported | NA | NA | NA |
| Cabré, 2018 | Continuous (Mean+SD) | Student- t test | NS | Categorical | Chi-square test or Fisher’s exact test | Sig |
| Cahir 2023 | Continuous (Mean+SD) | NA | NA | NA | NA | NA |
| Dechanont, 2021 | NA | NA | NA | Categorical | Chi-square analysis | Sig |
| Fattinger, 2000 | Continuous Median | Kruskal-Wallis comparison | NS | NA | NA | NA |
| Gebremariam 2014 | NA | NA | NA | Categorical | NA | NA |
| Giardina, 2018 | Continuous Median (IQR) | *U* Mann–Whitney test, | NS | Categorical | Bivariate analyses using regression | NS |
| Hopf, 2008 | Continuous Median | Mann–Whitney test | NS | NA | NA | NA |
| Jolivot, 2016 | Continuous Median (IQR) | Mann–Whitney test | NS | NA | NA | NA |
| Karuppannan, 2013 | NA | NA | NA | Categorical | Chi-square analysis | Sig |
| Komagamine 2024 | Continuous Median (IQR) | NA | NA | NA | NA | NA |
| Komagamine, 2019 | Continuous Median (IQR) | NA | NA | Categorical | Bivariate analyses using regression | NS |
| Kongkaew, 2013 | NA | NA | NA | Categorical | Pearson chi-squared test | Sig |
| Lagnaoui, 2000 | Continuous (Mean) | Kruskal-Wallis comparison | NS | NA | NA | NA |
| Lavan, 2019 | Continuous Median (IQR), range | Mann-Whitney U and Kruskal- Wallis tests | NS | NA | NA | NA |
| Laroche 2023 | Continuous Mean and Median | 95% confidence interval | Sig | Categorical | Chi-square analysis | Sig |
| Lea, 2019 | Continuous Median (range) | Mann-Whitney test | NS | NA | NA | NA |
| Leendertse, 2008 | Continuous Mean+SD | NA | NA | NA | NA | NA |
| Li 2021 | Continuous Median (IQR) | Mann-Whitney U | Sig | NA | NA | NA |
| Luttikhuis 2022 | Continuous Median (IQR) | NA | NA | NA | NA | NA |
| Marcum, 2012a | NA | NA | NA | Categorical | Chi-square analysis | NS |
| Marcum, 2012b | NA | NA | NA | Categorise (crude OR,95%cl) | Bivariate analyses using regression | NS |
| Marikova 2021 | Continuous Median | Mann-Whitney test | NS | NA | NA | NA |
| McLachlan, 2014 | Continuous Mean (range) | Student t-test | Sig | NA | NA | NA |
| Menéndez-Conde, 2011 | Continuous (Mean+SD) | NA | NA | Categorical | Chi-square analysis | NS |
| Mjörndal, 2002 | Continuous Median (range) | Wilcoxon sum | NS | NA | NA | NA |
| Mouton, 2016 | Continuous (Median (IQR) | NA | NA | NA | NA | NA |
| Ocovska 2022 | Continuous (Median (IQR) | NA | NA | NA | NA | NA |
| Olivier, 2002 | Continuous Mean+SD | No test | NA | NA | NA | NA |
| Olivier, 2009 | Continuous (Mean+SD) | Student *t* test | NS | Categorical | Chi-square analysis or Fisher’s Exact test | NS |
| Onder, 2002 | Sever ADR was Continuous (Mean+SD) | NA | NA | Categorical | Chi-square analysis | NS/ Sig with sever ADR only |
| Pedrós, 2014 | Continuous (Median) | Mann–Whitney test | Sig | Categorical | Chi-square analysis | Sig |
| Pedrós, 2016 | NA | NA | NA | Categorical | No association reported | NA |
| Pirmohamed, 2004 | Continuous (Median, interquartile range | Mann-Whitney U test | Sig | NA | NA | NA |
| Pourseyed, 2009 | Continuous Mean (rang) | Student *t* test | Sig | NA | NA | NA |
| Pouyanne, 2000 | NA | NA | NA | Categorical | Chi-square analysis | Sig |
| Santamaria-Pablos, 2009 | Continuous SD (95% CI) | Student *t* test | Sig | NA | NA | NA |
| Singh, 2011 | Continuous (Mean ± SD) | Not reported | Ns | NA | NA | NA |
| Smeaton, 2020 | Not clear | Bivariate analyses using regression | NS | Not clear | Not clear |  |
| Somers, 2010 | Continuous Mean | Not reported | Sig | NA | NA | NA |
| van der Hooft, 2008 | NA | NA | NA | Categorical | Confidence intervals (95%CI) were calculated | NS |
| Varallo, 2014 | NA | NA | NA | Categorical | Chi-square analysis | NS |
| von Euler, 2006 | Continuous Mean | NA | NA | NA | NA | NA |
| Wawruch, 2009 | NA | NA | NA | Categorical | Chi-square analysis | NS |

## Funnel plot 1:

The risk ratio of medication-related hospital admission by age category:

**
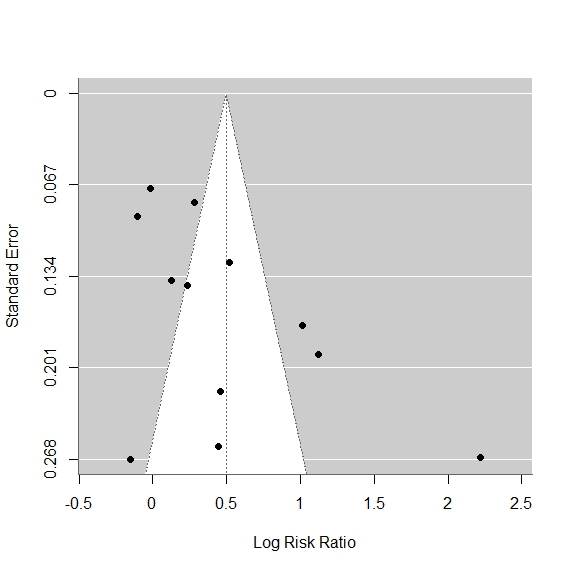
**

## Funnel plot 2:

The mean differences in age between the study groups:

**
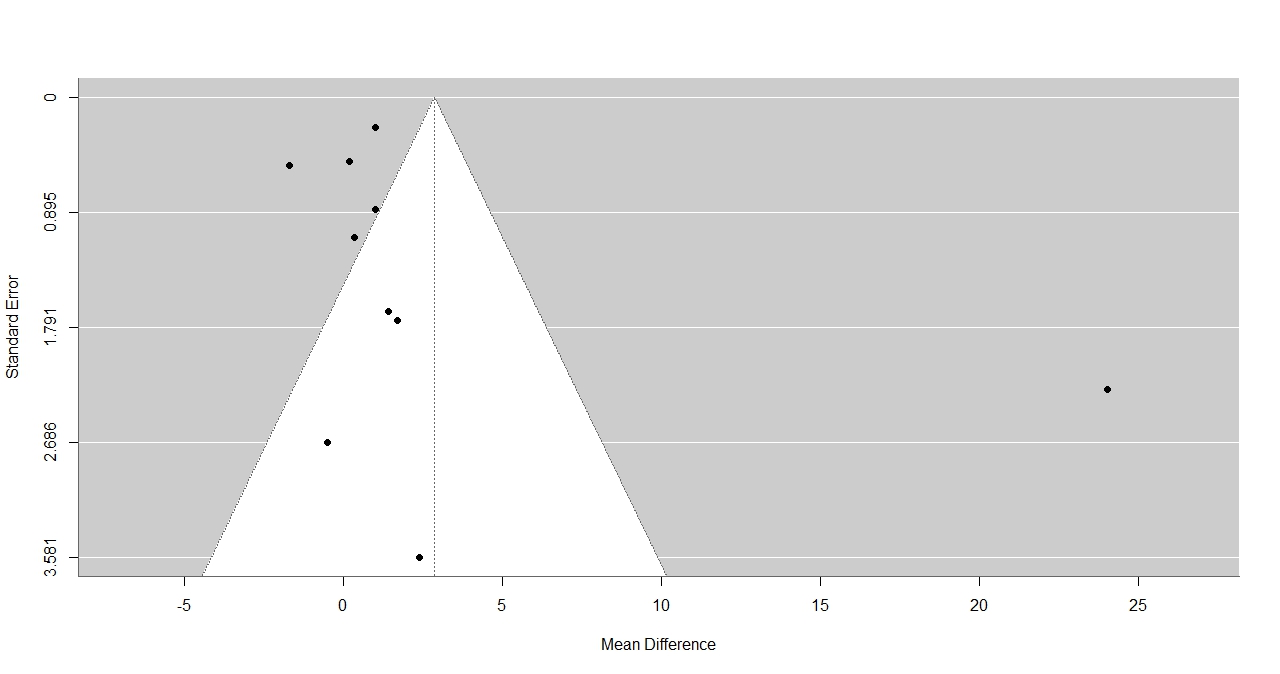
**

## Supplemental table 4. Certainty assessment

| \| **Certainty of evidence (GRADE)** \| \| --- \| | **Absolute effect** | \| **Relative effect (95% CI)** \| \| --- \| | \| **Study design** \| \| --- \| | \| **No. of studies** \| \| --- \| | \| **Outcome** \| \| --- \| |
| --- | --- | --- | --- | --- | --- | --- | --- | --- | --- | --- |
| **Very low**  **a,b,c,d,e,f*** | \| Higher odds of MRA among younger adults compared with older adults \| \| --- \| | \| RR 1.64 (1.15 to 2.35) \| \| --- \|  \|  \| \| --- \| | \| Observational studies \| \| --- \| | 12 | \| Association between age category and medication-related hospital admission (younger vs older) \| \| --- \| |
| **Very low**  **a,b,c,d,e,f**** | \| Patients with MRA were slightly older on average \| \| --- \| | \| MD 2.33 years (0.40 to 4.25) \| \| --- \| | Observational studies | 10 | \| Mean difference in age between study groups \| \| --- \| |

***** a. Risk of bias: downgraded one level due to potential residual confounding and variability in study quality, with several studies having unclear or heterogeneous outcome definitions.

b. Inconsistency: downgraded two levels due to substantial heterogeneity (I² = 96%) and wide variation in effect sizes across studies.

c. Indirectness: downgraded one level due to differences in the definition of medication-related hospital admission (e.g., WHO, Edwards and Aronson, study-specific, or unclear definitions).

d. Imprecision: not downgraded, as the confidence interval did not cross the line of no effect and the pooled estimate was statistically significant.

e. Publication bias: not downgraded due to uncertainty in interpretation of funnel plot asymmetry in the presence of high heterogeneity.

f. No upgrading factors were identified (e.g., no large magnitude of effect or clear dose–response relationship).

****** a. Downgraded for risk of bias due to observational design, variability in study quality, and potential residual confounding.
b. Downgraded for inconsistency due to substantial heterogeneity (I² = 93%) and variation in effect sizes across studies.
c. Downgraded for indirectness due to variability in definitions of medication-related hospital admission.
d. Downgraded for imprecision due to relatively wide confidence intervals.
e. Publication bias suspected based on funnel plot asymmetry; interpretation limited due to heterogeneity.
f. No upgrading factors identified.

## Supplementary table 5. The PRISMA 2020 checklist: An updated guideline for reporting systematic reviews.

| **Section and Topic** | **Item #** | **Checklist item** | **Location where item is reported** |
| --- | --- | --- | --- |
| **TITLE** | | |  |
| Title | 1 | Identify the report as a systematic review. | 1 |
| **ABSTRACT** | | |  |
| Abstract | 2 | See the PRISMA 2020 for Abstracts checklist. | Was applied, p2 |
| **INTRODUCTION** | | |  |
| Rationale | 3 | Describe the rationale for the review in the context of existing knowledge. | 3 |
| Objectives | 4 | Provide an explicit statement of the objective(s) or question(s) the review addresses. | 3 |
| **METHODS** | | |  |
| Eligibility criteria | 5 | Specify the inclusion and exclusion criteria for the review and how studies were grouped for the syntheses. | 4 |
| Information sources | 6 | Specify all databases, registers, websites, organisations, reference lists and other sources searched or consulted to identify studies. Specify the date when each source was last searched or consulted. | 3-4 |
| Search strategy | 7 | Present the full search strategies for all databases, registers and websites, including any filters and limits used. | In the supplement file |
| Selection process | 8 | Specify the methods used to decide whether a study met the inclusion criteria of the review, including how many reviewers screened each record and each report retrieved, whether they worked independently, and if applicable, details of automation tools used in the process. | 5 |
| Data collection process | 9 | Specify the methods used to collect data from reports, including how many reviewers collected data from each report, whether they worked independently, any processes for obtaining or confirming data from study investigators, and if applicable, details of automation tools used in the process. | 5-6 |
| Data items | 10a | List and define all outcomes for which data were sought. Specify whether all results that were compatible with each outcome domain in each study were sought (e.g. for all measures, time points, analyses), and if not, the methods used to decide which results to collect. | 4-6 |
|  | 10b | List and define all other variables for which data were sought (e.g. participant and intervention characteristics, funding sources). Describe any assumptions made about any missing or unclear information. | NA |
| Study risk of bias assessment | 11 | Specify the methods used to assess risk of bias in the included studies, including details of the tool(s) used, how many reviewers assessed each study and whether they worked independently, and if applicable, details of automation tools used in the process. | 5-6 |
| Effect measures | 12 | Specify for each outcome the effect measure(s) (e.g. risk ratio, mean difference) used in the synthesis or presentation of results. | 6 |
| Synthesis methods | 13a | Describe the processes used to decide which studies were eligible for each synthesis (e.g. tabulating the study intervention characteristics and comparing against the planned groups for each synthesis (item #5)). | NA |
|  | 13b | Describe any methods required to prepare the data for presentation or synthesis, such as handling of missing summary statistics, or data conversions. | NA |
|  | 13c | Describe any methods used to tabulate or visually display results of individual studies and syntheses. | 6 |
|  | 13d | Describe any methods used to synthesize results and provide a rationale for the choice(s). If meta-analysis was performed, describe the model(s), method(s) to identify the presence and extent of statistical heterogeneity, and software package(s) used. | 6 |
|  | 13e | Describe any methods used to explore possible causes of heterogeneity among study results (e.g. subgroup analysis, meta-regression). | 6 |
|  | 13f | Describe any sensitivity analyses conducted to assess robustness of the synthesized results. | NA |
| Reporting bias assessment | 14 | Describe any methods used to assess risk of bias due to missing results in a synthesis (arising from reporting biases). | NA |
| Certainty assessment | 15 | Describe any methods used to assess certainty (or confidence) in the body of evidence for an outcome. | 5 |
| **RESULTS** | | |  |
| Study selection | 16a | Describe the results of the search and selection process, from the number of records identified in the search to the number of studies included in the review, ideally using a flow diagram. | 7 |
|  | 16b | Cite studies that might appear to meet the inclusion criteria, but which were excluded, and explain why they were excluded. | NA |
| Study characteristics | 17 | Cite each included study and present its characteristics. | 8-12 |
| Risk of bias in studies | 18 | Present assessments of risk of bias for each included study. | In the supplement file |
| Results of individual studies | 19 | For all outcomes, present, for each study: (a) summary statistics for each group (where appropriate) and (b) an effect estimate and its precision (e.g. confidence/credible interval), ideally using structured tables or plots. | 14-20 |
| Results of syntheses | 20a | For each synthesis, briefly summarise the characteristics and risk of bias among contributing studies. | NA |
|  | 20b | Present results of all statistical syntheses conducted. If meta-analysis was done, present for each the summary estimate and its precision (e.g. confidence/credible interval) and measures of statistical heterogeneity. If comparing groups, describe the direction of the effect. | 21-25 |
|  | 20c | Present results of all investigations of possible causes of heterogeneity among study results. | NA |
|  | 20d | Present results of all sensitivity analyses conducted to assess the robustness of the synthesized results. | 21-22 |
| Reporting biases | 21 | Present assessments of risk of bias due to missing results (arising from reporting biases) for each synthesis assessed. | NA |
| Certainty of evidence | 22 | Present assessments of certainty (or confidence) in the body of evidence for each outcome assessed. | NA |
| **DISCUSSION** | | |  |
| Discussion | 23a | Provide a general interpretation of the results in the context of other evidence. | 26 |
|  | 23b | Discuss any limitations of the evidence included in the review. | 26-27 |
|  | 23c | Discuss any limitations of the review processes used. | 27-28 |
|  | 23d | Discuss implications of the results for practice, policy, and future research. | 28 |
| **OTHER INFORMATION** | | |  |
| Registration and protocol | 24a | Provide registration information for the review, including register name and registration number, or state that the review was not registered. | 3 |
|  | 24b | Indicate where the review protocol can be accessed, or state that a protocol was not prepared. | NA |
|  | 24c | Describe and explain any amendments to information provided at registration or in the protocol. | NA |
| Support | 25 | Describe sources of financial or non-financial support for the review, and the role of the funders or sponsors in the review. | NA |
| Competing interests | 26 | Declare any competing interests of review authors. | NA |
| Availability of data, code and other materials | 27 | Report which of the following are publicly available and where they can be found: template data collection forms; data extracted from included studies; data used for all analyses; analytic code; any other materials used in the review. | √ |

*From:*  Page MJ, McKenzie JE, Bossuyt PM, Boutron I, Hoffmann TC, Mulrow CD, et al. The PRISMA 2020 statement: an updated guideline for reporting systematic reviews. BMJ 2021;372:n71. doi: 10.1136/bmj

## References:

1. Ahern F, Sahm LJ, Lynch D et al. Determining the frequency and preventability of adverse drug reaction-related admissions to an Irish University Hospital: a cross-sectional study. Emerg Med J. 2014;31(1):24-9.

2. Alexopoulou A, Dourakis SP, Mantzoukis D et al. Adverse drug reactions as a cause of hospital admissions: a 6-month experience in a single center in Greece. Eur J Intern Med. 2008;19(7):505-10.

3. Alvarez PA, Bril F, Castro V et al. Adverse drug reactions as a reason for admission to an internal medicine ward in Argentina. Int J Risk Saf Med. 2013;25(3):185-92.

4. Angamo MT, Curtain CM, Chalmers L et al. Predictors of adverse drug reaction-related hospitalisation in Southwest Ethiopia: A prospective cross-sectional study. PLoS One. 2017;12(10):e0186631.

5. Asio L, Nasasira M, Kiguba R. Hospital admissions attributed to adverse drug reactions in tertiary care in Uganda: burden and contributing factors. Therapeutic Advances in Drug Safety. 2023;14:20420986231188842.

6. Cabré M, Elias L, Garcia M et al. Avoidable hospitalizations due to adverse drug reactions in an acute geriatric unit. Analysis of 3,292 patients. Medicina Clínica (English Edition). 2018;150(6):209-14.

7. Cahir C, Curran C, Walsh C et al. Adverse drug reactions in an ageing PopulaTion (ADAPT) study: Prevalence and risk factors associated with adverse drug reaction-related hospital admissions in older patients. Front Pharmacol. 2023;13:1029067.

8. Gebremariam SN, Sema FD, Jara AG et al. Medication-Related Hospital Admission Among Patients Admitted to the Emergency Ward at the University of Gondar, North West Ethiopia: A Cross Sectional Study. Drug, Healthcare and Patient Safety. 2024:75-88.

9. Giardina C, Cutroneo PM, Mocciaro E et al. Adverse Drug Reactions in Hospitalized Patients: Results of the FORWARD (Facilitation of Reporting in Hospital Ward) Study. Front Pharmacol. 2018;9(350). <https://doi.org/10.3389/fphar.2018.00350>.

10. Karuppannan M, Nee TK, Ali SM et al. The prevalence of adverse drug event-related admissions at a local hospital in Malaysia. Archives of Pharmacy Practice. 2013;4(4).

11. Komagamine J. Prevalence of urgent hospitalizations caused by adverse drug reactions: a cross-sectional study. Sci Rep. 2024;14(1):6058.

12. Komagamine J, Kobayashi M. Prevalence of hospitalisation caused by adverse drug reactions at an internal medicine ward of a single centre in Japan: a cross-sectional study. BMJ open. 2019;9(8):e030515.

13. Laroche ML, Gautier S, Polard E et al. Incidence and preventability of hospital admissions for adverse drug reactions in France: A prospective observational study (IATROSTAT). Br J Clin Pharmacol. 2023;89(1):390-400.

14. Lavan AH, O'Mahony D, Buckley M et al. Adverse drug reactions in an oncological population: prevalence, predictability, and preventability. The oncologist. 2019;24(9):e968.

15. Lea M, Mowe M, Mathiesen L et al. Prevalence and risk factors of drug-related hospitalizations in multimorbid patients admitted to an internal medicine ward. PLoS One. 2019;14(7):e0220071.

16. Li R, Curtis K, Zaidi STR et al. Prevalence, characteristics, and reporting of adverse drug reactions in an Australian hospital: a retrospective review of hospital admissions due to adverse drug reactions. Expert Opin Drug Saf. 2021;20(10):1267-74.

17. Maříková M, Očovská Z, Nerad V et al. Hospital admissions to geriatric ward related to adverse drug events: a cross-sectional study from the Czech Republic. Int J Clin Pharm. 2021;43(5):1218-26.

18. McLachlan C, Yi M, Ling A et al. Adverse drug events are a major cause of acute medical admission. Intern Med J. 2014;44(7):633-8.

19. Menéndez-Conde CP, Vicedo TB, Silveira ED et al. Adverse drug reactions which provoke hospital admission. Farmacia Hospitalaria (English Edition). 2011;35(5):236-43.

20. Mjörndal T, Boman MD, Hägg S et al. Adverse drug reactions as a cause for admissions to a department of internal medicine. Pharmacoepidemiol Drug Saf. 2002;11(1):65-72.

21. Mouton JP, Njuguna C, Kramer N et al. Adverse drug reactions causing admission to medical wards: A cross-sectional survey at 4 hospitals in South Africa. Medicine. 2016;95(19).

22. Očovská Z, Maříková M, Kočí J et al. Drug-related hospital admissions via the department of emergency medicine: A cross-sectional study from the Czech republic. Front Pharmacol. 2022;13:899151.

23. Olivier P, Bertrand L, Tubery M et al. Hospitalizations because of adverse drug reactions in elderly patients admitted through the emergency department. Drugs Aging. 2009;26(6):475-82.

24. Onder G, Pedone C, Landi F et al. Adverse drug reactions as cause of hospital admissions: results from the Italian Group of Pharmacoepidemiology in the Elderly (GIFA). J Am Geriatr Soc. 2002;50(12):1962-8.

25. Pedrós C, Quintana B, Rebolledo M et al. Prevalence, risk factors and main features of adverse drug reactions leading to hospital admission. Eur J Clin Pharmacol. 2014;70(3):361-7.

26. Pedrós C, Formiga F, Corbella X et al. Adverse drug reactions leading to urgent hospital admission in an elderly population: prevalence and main features. Eur J Clin Pharmacol. 2016;72(2):219-26.

27. Pourseyed S, Fattahi F, Pourpak Z et al. Adverse drug reactions in patients in an Iranian department of internal medicine. Pharmacoepidemiol Drug Saf. 2009;18(2):104-10.

28. Pouyanne P, Haramburu F, Imbs JL et al. Admissions to hospital caused by adverse drug reactions: cross sectional incidence study. BMJ. 2000;320(7241):1036.

29. Santamaria-Pablos A, Redondo-Figuero C, Baena M et al. Negative results related to drugs required in hospitalisation. Farmacia Hospitalaria (English Edition). 2009;33(1):12-25.

30. Singh H, Kumar BN, Sinha T et al. The incidence and nature of drug-related hospital admission: a 6-month observational study in a tertiary health care hospital. J Pharmacol Pharmacother. 2011;2(1):17.

31. Somers A, Robays H, Vander Stichele R et al. Contribution of drug related problems to hospital admission in the elderly. J Nutr Health Aging. 2010;14(6):477-82.

32. Varallo FR, Capucho HC, da Silva Planeta C et al. Possible adverse drug events leading to hospital admission in a Brazilian teaching hospital. Clinics. 2014;69(3):163-7.

33. Wawruch M, Zikavska M, Wsolova L et al. Adverse drug reactions related to hospital admission in Slovak elderly patients. Arch Gerontol Geriatr. 2009;48(2):186-90.

34. Adedapo AD, Adedeji WA, Adedapo IA et al. Cohort study on adverse drug reactions in adults admitted to the medical wards of a tertiary hospital in Nigeria: Prevalence, incidence, risk factors and fatality. Br J Clin Pharmacol. 2021;87(4):1878-89.

35. Brvar M, Fokter N, Bunc M et al. The frequency of adverse drug reaction related admissions according to method of detection, admission urgency and medical department specialty. BMC Clin Pharmacol. 2009;9(1):1-8.

36. Dechanont S, Jedsadayanmata A, Butthum B et al. Hospital admissions associated with medication-related problems in Thai older patients: a multicenter prospective observational study. Journal of patient safety. 2021;17(1):15-22.

37. Fattinger K, Roos M, Vergères P et al. Epidemiology of drug exposure and adverse drug reactions in two Swiss departments of internal medicine. Br J Clin Pharmacol. 2000;49(2):158-67.

38. Kongkaew C, Hann M, Mandal J et al. Risk factors for hospital admissions associated with adverse drug events. Pharmacotherapy: The Journal of Human Pharmacology and Drug Therapy. 2013;33(8):827-37.

39. Lagnaoui R, Moore N, Fach J et al. Adverse drug reactions in a department of systemic diseases-oriented internal medicine: prevalence, incidence, direct costs and avoidability. Eur J Clin Pharmacol. 2000;56(2):181-6.

40. Luttikhuis H, Blomaard L, van der Kaaij M et al. Geriatric characteristics and the risk of drug-related hospital admissions in older Emergency Department patients. Eur Geriatr Med. 2022:1-9.

41. Marcum ZA, Pugh MJV, Amuan ME et al. Prevalence of potentially preventable unplanned hospitalizations caused by therapeutic failures and adverse drug withdrawal events among older veterans. Journals of Gerontology Series A: Biomedical Sciences and Medical Sciences. 2012;67(8):867-74.

42. Marcum ZA, Amuan ME, Hanlon JT et al. Prevalence of unplanned hospitalizations caused by adverse drug reactions in older veterans. J Am Geriatr Soc. 2012;60(1):34-41.

43. Pirmohamed M, James S, Meakin S et al. Adverse drug reactions as cause of admission to hospital: prospective analysis of 18 820 patients. BMJ. 2004;329(7456):15-9.

44. van der Hooft CS, Dieleman JP, Siemes C et al. Adverse drug reaction‐related hospitalisations: a population‐based cohort study. Pharmacoepidemiol Drug Saf. 2008;17(4):365-71.

45. von Euler M, Eliasson E, Öhlén G et al. Adverse drug reactions causing hospitalization can be monitored from computerized medical records and thereby indicate the quality of drug utilization. Pharmacoepidemiol Drug Saf. 2006;15(3):179-84.

46. Jolivot P-A, Pichereau C, Hindlet P et al. An observational study of adult admissions to a medical ICU due to adverse drug events. Annals of intensive care. 2016;6(1):1-12.

47. Leendertse AJ, Egberts AC, Stoker LJ et al. Frequency of and risk factors for preventable medication-related hospital admissions in the Netherlands. Arch Intern Med. 2008;168(17):1890-6.

48. Olivier P, Boulbés O, Tubery M et al. Assessing the feasibility of using an adverse drug reaction preventability scale in clinical practice. Drug Saf. 2002;25(14):1035-44.

49. Hopf Y, Watson M, Williams D. Adverse-drug-reaction related admissions to a hospital in Scotland. Pharm World Sci. 2008;30(6):854.

50. Smeaton T, McElwaine P, Cullen J et al. A prospective observational pilot study of adverse drug reactions contributing to hospitalization in a cohort of middle-aged adults aged 45–64 years. Drugs & Therapy Perspectives. 2020;36(3):123-30.
